# Global Diversity and Phylogeny of Pelagic Shrimps of the Former Genera *Sergestes* and *Sergia* (Crustacea, Dendrobranchiata, Sergestidae), with Definition of Eight New Genera

**DOI:** 10.1371/journal.pone.0112057

**Published:** 2014-11-19

**Authors:** Alexander L. Vereshchaka, Jørgen Olesen, Anastasia A. Lunina

**Affiliations:** 1 Institute of Oceanology of Russian Academy of Sciences, Russia, Moscow; 2 Natural History Museum of Denmark, University of Copenhagen, Copenhagen, Denmark; Dauphin Island Sea Lab, United States of America

## Abstract

We revise the global diversity of the former genera *Sergia* and *Sergestes* which include 71 valid species. The revision is based on examination of more than 37,000 specimens from collections in the Natural History Museum of Denmark and the Museum of Natural History, Paris. We used 72 morphological characters (61 binary, 11 multistate) and *Sicyonella antennata* as an outgroup for cladistic analysis. There is no support for the genera *Sergia* and *Sergestes* as they have been defined until now. We define and diagnose eight genera of the former genus *Sergia* (*Sergia* and new genera *Gardinerosergia*, *Phorcosergia*, *Prehensilosergia*, *Robustosergia*, *Scintillosergia*, *Challengerosergia*, and *Lucensosergia*) and seven genera of the former genus *Sergestes* (*Sergestes, Deosergestes*, *Eusergestes*, *Allosergestes*, *Parasergestes, Neosergestes*, and a new genus *Cornutosergestes*). An identification key is presented for all genera of the family Sergestidae. The phylogeny of Sergestidae is mainly based on three categories of characters related to: (1) general decapod morphology, (2) male copulatory organs, and (3) photophores. Only simultaneous use of all three character types resulted in a resolved tree with minimal Bootstrap support 75 for each clade. Most genera are interzonal mesopelagic migrants, some are benthopelagic (*Scintillosergia, Lucensosergia*), bathypelagic (*Sergia*), or epipelagic (*Cornutosergestes*). Within each of meso- and benthopelagic genera there is one species with panoceanic distribution, while most species ranges are restricted to a single ocean. The genera demonstrate two different strategies expressed both in morphology and behavior: protective (*Eusergestes, Sergestes, Cornutosergestes, Prehensilosergia, Scintillosergia, Lucensosergia, Challengerosergia, Gardinerosergia, Robustosergia, Phorcosergia*, *Sergia*) and offensive (*Neosergestes, Parasergestes, Allosergestes, Deosergestes*).

## Introduction

**Figure 1:**
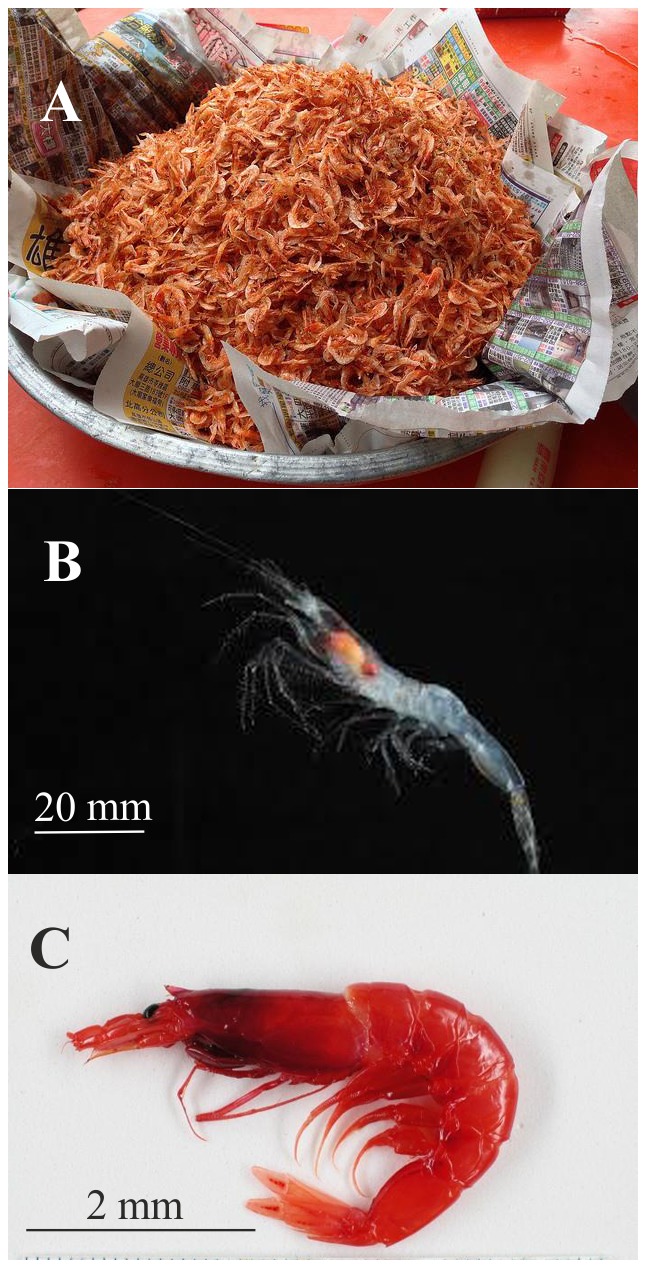
View of *Lucensosergia lucens* at fish market, Suruga Bay, Japan (A), *Deosergestes* sp. (B) and *Robustosergia robusta* (C) from midwater of the North Atlantic.

The decapod suborder Dendrobranchiata (Crustacea, Malacostraca) includes shrimps that have an important role both ecologically and economically in marine and estuarine ecosystems. The approximately 500 extant species range from shallow waters in the tropics to depths of about 1000 m on the continental slopes [1]. Species of the Sergestidae are among the most common in many ecosystems [2] – [3] and important objects of fisheries in some areas, such as *Sergestes lucens* in Japan (Fig. 1a). Despite their importance, the sergestids are still poorly understood with regard to higher level classification and phylogenetic relationships.

The two genera, *Sergestes* H. Milne-Edwards, 1830 (Fig 1b) and *Sergia* Stimpson, 1860 (Fig. 1c), together most certainly form a monophyletic group based on a number of synapomorphies such as the presence of an organ of Pesta and dermal photophores [4] – [8] and comprise 71 species, i.e. two-thirds of all known recent sergestids. The taxonomic status of *Sergestes* and *Sergia* and the phylogenetic relationship of their constituent species are the object of the present paper. The taxonomic history of *Sergestes* and *Sergia* goes back to the first half of the nineteenth century when H. Milne-Edwards [9] and Stimpson [10] described *Sergestes* and *Sergia,* respectively. Among later researchers, the most productive and important was H. J. H. Hansen [4], [11]–[17], who critically reviewed the existing knowledge and generated much new data on sergestid taxonomy and classification. Hansen [4], [11] synonymized *Sergia* with *Sergestes*, which he later subdivided into two species groups [12], [14]. Yaldwyn [5] synthesised the available information about the taxonomy and morphology of *Sergestes* (sensu Hansen [4], [11]), dividing the genus into two subgenera, *Sergestes* and *Sergia*, and nine species groups (six in *Sergestes* and three in *Sergia*). Later researchers mostly followed Yaldwyn's classification at least until the mid-1970s, when Omori [6] reviewed the differences between Yaldwyn's subgenera *Sergestes* and *Sergia* both with respect to morphology and ontogeny and raised their taxonomic status to the generic level. Most recent authors have followed the separation of the two genera (for example, [7] – [8], [18]).

As a result of the work of Vereshchaka [7] – [8], [19], which involved the checking of all available type material and documenting of intra- and interspecific variation of many characters, six species were synonymized and 13 new species were recognized, yielding a total number of 71 species in *Sergestes* and *Sergia.*

During this revisionary work it was noted that both *Sergia* and *Sergestes* contained taxonomic groups that appeared sufficiently well-defined to be treated as genera, but such a step was at the time postponed until it had been tested by a more comprehensive phylogenetic analysis. Recently, Judkins and Kensley [18] erected five new genera of *Sergestes* replacing Yaldwyn's species groups. They provided diagnoses for the new genera mostly based on the characters from Yaldwyn's work but provided no new evidence for the support of the new taxa.

Previous attempts to classify sergestids above the species level have focused on various subsets of characters (e.g., male copulatory organs or photophores) which have yielded conflicting phylogenetic and classificatory signals [7] – [8].

In this paper we present a phylogenetic approach based upon extensive studies of most available specimens and using all character systems (general external morphology, copulatory organs such as the petasma and clasping organ in males, and photophores). We use the resulting phylogeny to present a new classification, and to explore morphological, ecological, and biogeographical patterns within the clades.

## Materials and Methods

### 1. Material examined

This study is primarily based on material in the Natural History Museum of Denmark (former ‘Zoological Museum, University of Copenhagen’) and Museum National d'Histoire Naturelle (Paris), which hold the richest collections of sergestids in the world. In Copenhagen the primary contribution of sergestids came from the expeditions “Dana-1” and “Dana-2” (Table 1). In Paris the primary contribution came from “Caride I–V” and “Cyclone 3–6”, specimen numbers for studied samples have not established. Much of these two museums' sergestid material was studied by Vereshchaka [7] – [8], and more than 28,000 specimens of *Sergestes* and 9,000 specimens of *Sergia* were examined; much information about the examined material such as distribution and information about type specimens can also be found in Vereshchaka's papers [7] – [8].

**Table 1:** Type material with ZMUC identification numbers used in the studies.

Genus sensu Junkins and Kensley (2008) [18]	Genus in this paper	Species names	Collection number
Allosergestes	Allosergestes	index	CRU-001621
		oleseni	CRU-004840
		verpus	CRU-009525, CRU-020205
		vinogradovi	CRU-004838, CRU-004839
Deosergestes	Deosergestes	coalitus	CRU-001619, CRU-004532, CRU-004546, CRU-009526
		corniculum	CRU-004522, CRU-006077
		disjunctus	CRU-004535, CRU-001618
		rubroguttatus	CRU-004518
		seminudus	CRU-008051
Eusergestes	Eusergestes	antarcticus	CRU-004834, CRU-004835
		arcticus	CRU-005590, CRU-007960, CRU-009528
Neosergestes	Neosergestes	armatus	CRU-005626
		consobrinus	CRU-004550
		edwardsii	CRU-006329, CRU-005879, CRU-007619
		orientalis	CRU-007649
		semissis	CRU-001624
		tantillus	CRU-001623
Parasergestes	Parasergestes	cylindricus	CRU-004527
		sirenkoi	CRU-004841, CRU-004842
		stimulator	CRU-001622
Sergestes	Cornutosergestes	cornutus	CRU-004533, CRU-006083
		mepae	CRU-004836, CRU-004837
	Sergestes	atlanticus	CRU-006470
Sergia	Challengerosergia	fulgens	CRU-006472
		hansjacobi	CRU-001610, CRU-001611
		jeppeseni	CRU-003614
		oksanae	CRU-003615, CRU-003616
		stellata	CRU-001607
	Gardinerosergia	bigemmea	CRU-001627
		inaequalis	CRU-001628, CRU-001604
		kensleyi	CRU-003605, CRU-003606, CRU-003619
	Lucensosergia	crosnieri	CRU-003617, CRU-003618
	Phorcosergia	burukovskii	CRU-003607, CRU-003608, CRU-003609
		filicta	CRU-001603
		maxima	CRU-001609, CRU-001625
		potens	CRU-001608, CRU-001626
		wolffi	CRU-001612, CRU-001613
	Robustosergia	extenuata	CRU-001602
		vityazi	CRU-003610, CRU-003611, CRU-003612
	Scintillosergia	scintillans	CRU-001606
	Sergia	laminata	CRU-001605
		tenuiremis	CRU-008362, CRU-009527

### 2. Morphology and Characters

The morphological information provided by Vereshchaka [7] – [8] forms the basis of the phylogenetic analysis of *Sergestes* and *Sergia* presented here. Seventy-one species were included in the analysis, 36 belonging to the former *Sergestes,* 35 to the former *Sergia* (Table 2). Seventy-two morphological characters were identified (61 binary, 11 multistate). The characters used in this study fall into three main categories: 24 general external characters, 26 characters related to male copulatory organs, and 22 characters related to photophore structures.

**Table 2:** Names of old and new taxa within Sergestes sensu Hansen (1903; 1919) [4], [13].

Subgenera sensu Burkenroad (1937, 1945) [23], [43] and Yaldwyn (1957) [5], equivalent to genera sensu Omori (1974) [6]	Species groups sensu Yaldwyn (1957) [5]	Species groups sensu Vereshchaka (2000; 2009) [7] – [8]	Genus sensu Junkins and Kensley (2008) [18]	Genus in this paper	Species included
Sergestes	“Sergestes arcticus”	“Sergestes arcticus”	Eusergestes	Eusergestes	antarcticus arcticus
					similis
	“Sergestes atlanticus”	“Sergestes atlanticus”	Sergestes	Sergestes	atlanticus
		“Sergestes cornutus”		**Cornutosergestes**	cornutus
					mepae
	“Sergestes edwardsii”	“Sergestes edwardsii”	Neosergestes	Neosergestes	brevispinatus
					consobrinus
					edwardsii
					orientalis
					semissis
					tantillus
	“Sergestes vigilax”	“Sergestes vigilax”	Parasergestes	Parasergestes	armatus
					cylindricus
					diapontius
					halia
					sirenkoi
					stimulator
					vigilax
	“Sergestes corniculum”	“Sergestes corniculum”	Deosergestes	Deosergestes	coalitus
					corniculum
					disjunctus
					henseni
					paraseminudus
					pediformis
					rubroguttatus
					seminudus
	“Sergestes sargassi”	“Sergestes sargassi”	Allosergestes	Allosergestes	index
					nudus
					oleseni
					pectinatus
					pestafer
					sargassi
					verpus
					vinogradovi
Sergia	“Sergestes challengeri”	“Sergia prehensilis”	-	**Prehensilosergia**	prehensilis
				**Scintillosergia**	scintillans
		“Sergia lucens ”		**Lucensosergia**	crosnieri
					erythraeensis
					foresti
					lucens
		“Sergia challengeri”		**Challengerosergia**	challengeri
					fulgens
					hansjabobi
					jeppeseni
					oksanae
					stellata
					talismani
					umitakae
	“Sergestes robustus”	“Sergia gardineri”		**Gardinerosergia**	bigemmea
					gardineri
					inequalis
					kensleyi
					splendens
		“Sergia robusta”		**Robustosergia**	extenuata
					regalis
					robusta
					vityazi
		“Sergia phorca”		**Phorcosergia**	bisulcata
					burukovskii
					filicta
					grandis
					maxima
					phorca
					plumea
					potens
					wolffi
	“Sergestes japonicus”	“Sergia tenuiremis”		Sergia	tenuiremis
		“Sergia inoa”			inoa
		“Sergia japonica”			japonica
					laminata

New genera are in bold.

1. General external characters relate to external morphology such as the feeding/catching limbs (mainly maxilliped III, pereopods I–III). In general, external morphology shows little diversity.
2. Male copulatory organs include the petasma and the clasping organ (modified part of Antenna I). The petasma is a particularly complicated structure which exhibits much variation; for example, the petasma is sometimes very different in species that are otherwise very similar (closely related), or, vice versa, very similar in species that are otherwise very different (distantly related).
3. Photophores. These organs are present in most species of the genus *Sergia*. In photophores, a lens may be absent or present. A challenge in using photophores is that they fade away in alcohol preserved material. Reliable information can often only be obtained for flat and transparent body parts (i.e. scaphocerites and uropods).

**Figure 2:**
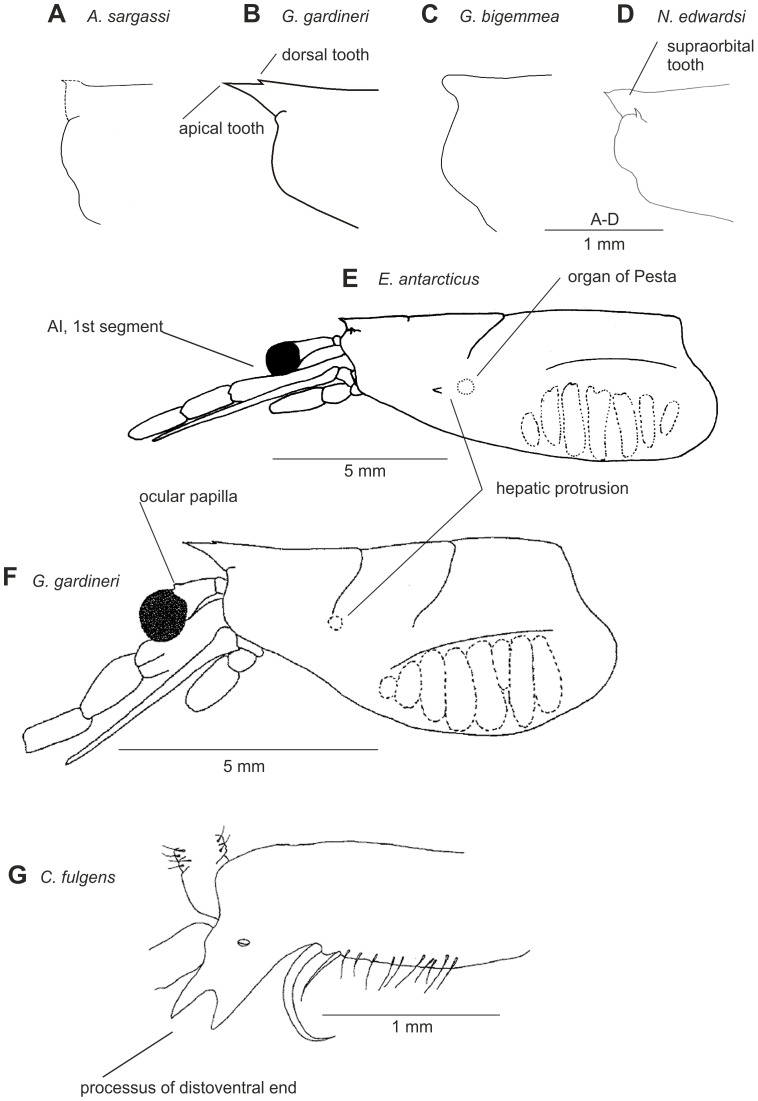
General morphological characters: rostrum (A–D), carapace (E–F), ocular papilla (F), peduncle of Antenna I (G).

**Figure 3:**
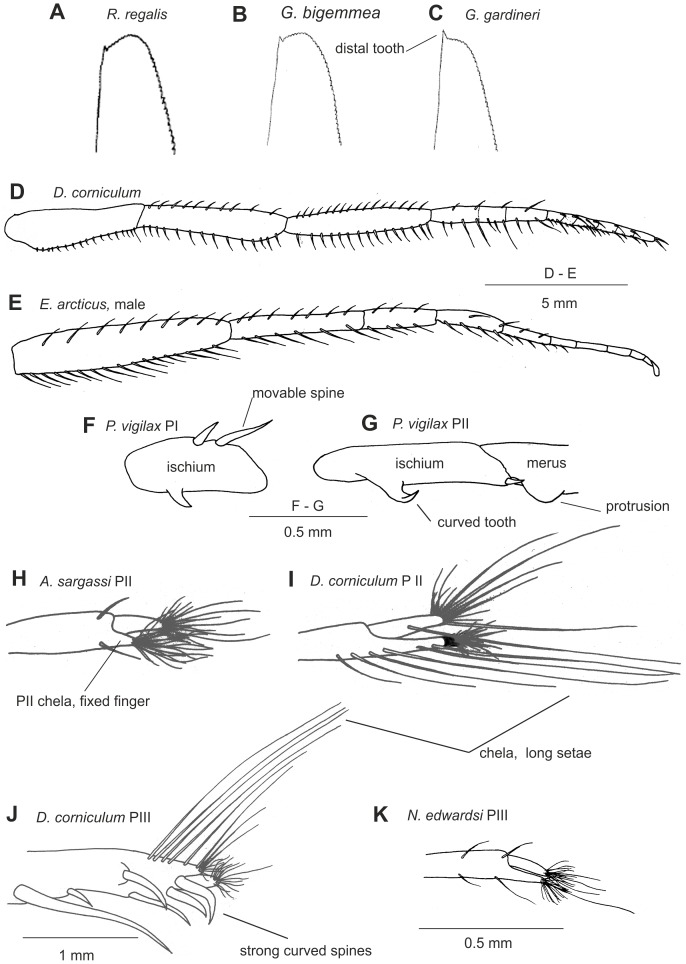
General morphological characters: scaphocerite (A–C), maxilliped III (D–E), ischium of pereopod I (F), ischium and merus of pereopod II (G), chelae of pereopod II (H, I), chelae of pereopod III (J, K).

**Figure 4:**
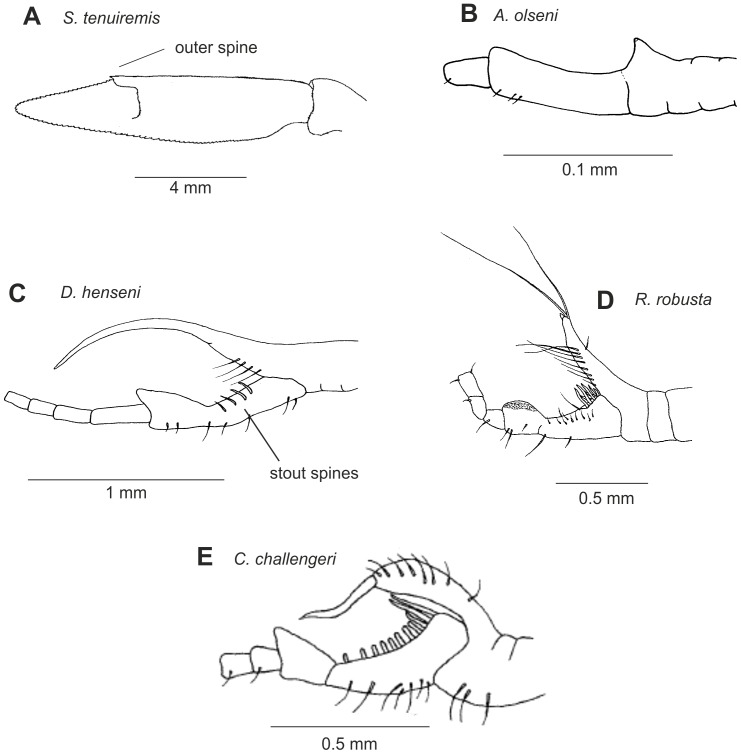
Morphological characters: uropodal exopod (A) and male clasping organ (B–E).

**Figure 5:**
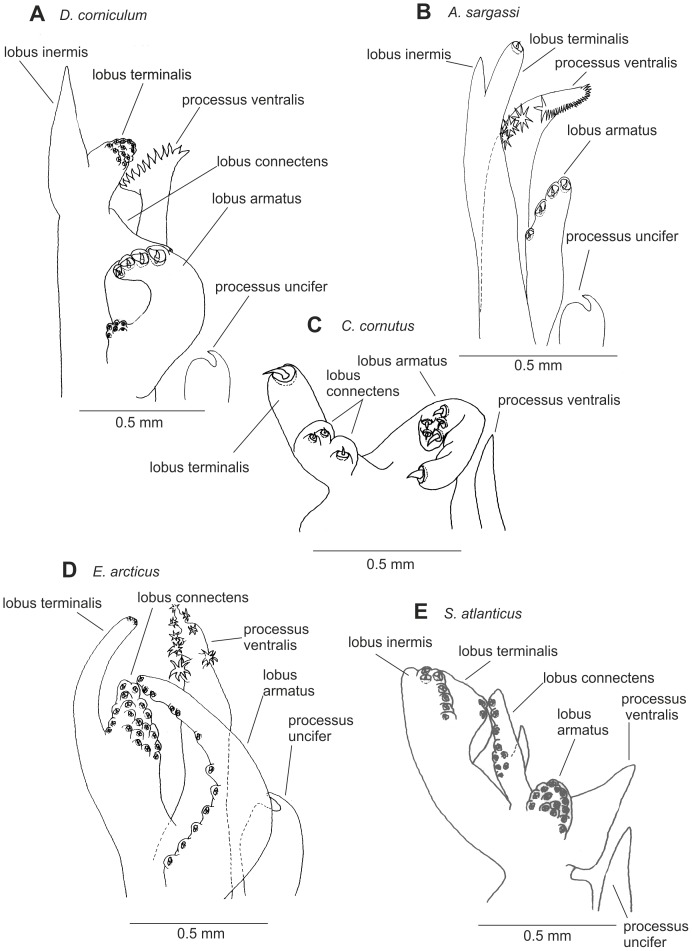
General morphology of petasma: *Deosergestes* (A), *Allosergestes* (B), *Cornutosergestes* (C), *Eusergestes* (D), and *Sergestes* (E).

**Figure 6:**
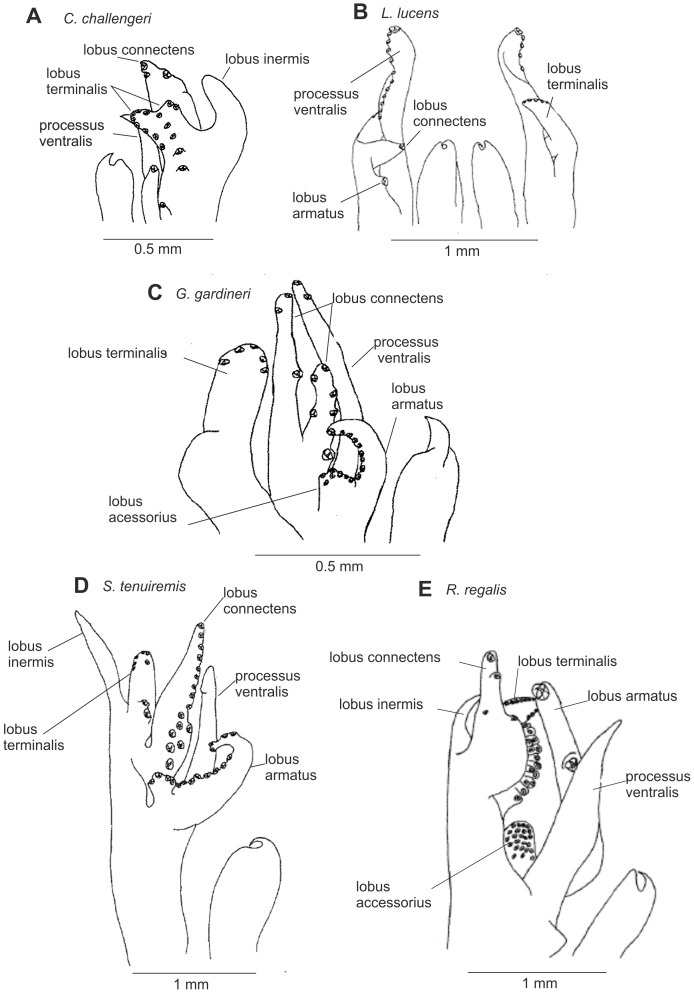
General morphology of petasma: *Challengerosergia* (A), *Lucensosergia* (B), *Gardinerosergia* (C), *Sergia* (D), and *Robustosergia* (E).

**Figure 7:**
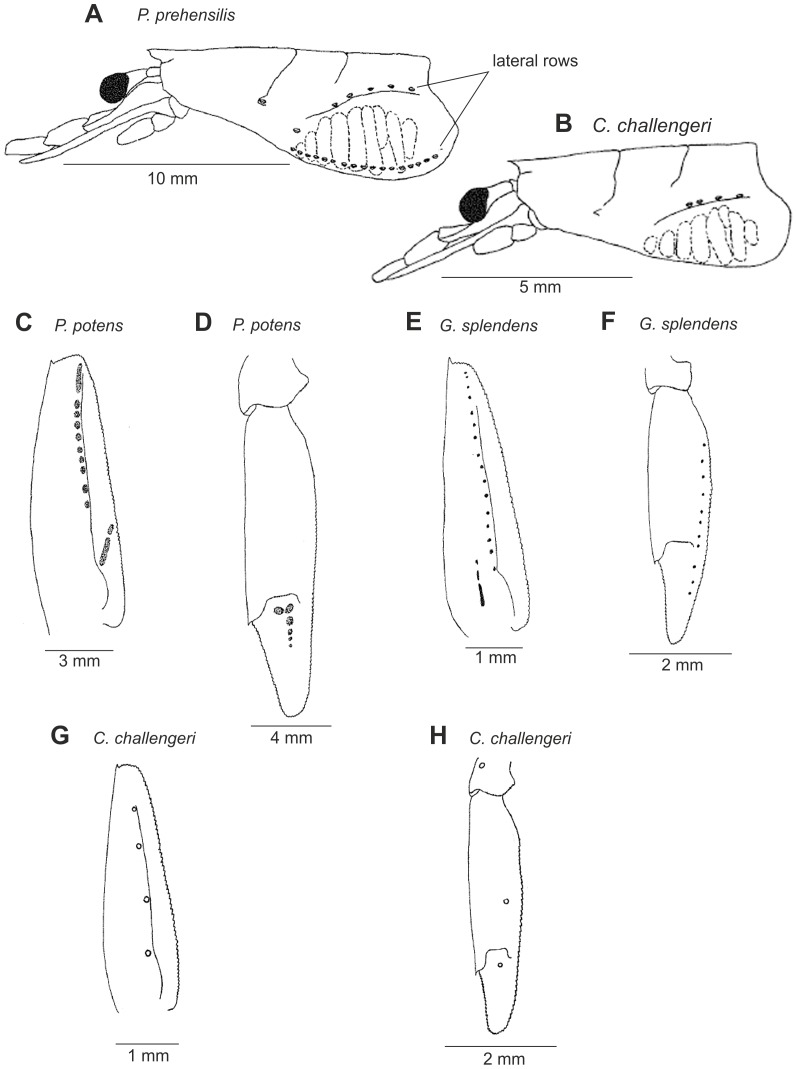
Dermal photophores: carapace of *Prehensilosergia* (A), carapace of *Challengerosergia* (B), scaphocerite of *Phorcosergia* (C), uropodal exopod of *Phorcosergia* (D), scaphocerite of *Gardinerosergia* (E), uropodal exopod of *Gardinerosergia* (F), scaphocerite of *Challengerosergia* (G), uropodal exopod of *Challengerosergia* (H).

Petasmas and photophores are the two classical organ systems used in the classification and systematics of the Sergestidae. However, in many cases photophore arrangements and petasma structure are incongruent. Species having similar photophore arrangements may have different petasmas and vice versa. This will be explored further below. Detailed information on the morphology underlying the characters used in this work can be found in [7] – [8], but some of the key characters are illustrated here (Figs 2–7). Our terminology follows [7] – [8].

### 3. Anatomical Abbreviations

We label most general anatomical characters directly on the figures but structures relating to the petasma, are abbreviated as follows:

LA − lobus armatus

LAc − lobus accessorius

LC − lobus connectens

LI − lobus inermis

LT − lobus terminalis

PU − processus uncifer

PV − processus ventralis

Description of characters and their states is presented in Table 3.

**Table 3:** List of morphological characters and their states.

Character No	Character state	State No	Reference to figure	
**CARAPACE**				
0	Integument firm	0		
	Integument membranous	1		
1	Frontal margin oblique	0	2B,D	
	Frontal margin vertical	1	2A	
2	Supraorbital tooth absent	0	2A–C	
	Supraorbital tooth present	1	2D	
3	Hepatic protrusion forming a barb	0	2F	
	Hepatic protrusion forming a spine	1	2E	
	Hepatic protrusion absent	2		
**EYE**				
4	Ocular papilla absent or rudimentary, <0.3 times as long as wide at base in dorsal view	0	2E	
	Ocular papilla moderately developed (0.3–0.6 times as long as wide)	1	2E	
	Ocular papilla much developed (>0.6 times as long as wide)	2		
**ANTENNULA**				
5	First segment elongate, ≥1.5 times as long as third segment	0	2E	
	First segment short, <1.5 times as long as third segment	1	2F	
6	Third segment lacking distoventral processus in male	0		
	Third segment bearing distoventral processus in male	1	2G	
**ANTENNA**				
7	Distal tooth of scaphocerite rudimentary, not reaching distal end of blade	0	3A	
	Distal tooth of scaphocerite developed, reaching or overreaching distal end of blade	1	3B,C	
**FIRST MAXILLIPED**				
8	Endopod developed, divided into 3–4 segments	0		
	Endopod reduced, divided into 2 segments	1		
**THIRD MAXILLIPED**				
9	Moderately developed, <2.0 times as long as first pereopod	0	2E	
	Elongated,>2.0 times as long as first pereopod	1	2F	
10	Not sexually dimorphic, dactyl not modified	0	3D	
	Sexually dimorphic, dactyl modified in males	1	3E	
11	Not subdivided into specialized subsegments	0	2E	
	Subdivided into specialized subsegments	1	2F	
12	Dactyl consists 4 specialized subsegments	0		
	Dactyl consists 5 specialized subsegments	1		
	Dactyl consists 6 specialized subsegments	2		
	Dactyl consists 7 specialized subsegments	3		
**FIRST PEREOPOD**				
13	Ischium lacking strong movable spines	0		
	Ischium bearing strong movable spines	1	3F	
**SECOND PEREOPOD**				
14	Ischium lacking strong distally curved tooth	0		
	Ischium bearing strong distally curved tooth	1	3G	
15	Merus lacking distal protrusion	0		
	Merus bearing distal protrusion	1	3G	
16	Fixed finger in chela rudimentary, shorter then dactyl	0	3H	
	Fixed finger developed, as long as dactyl	1	3I	
17	Chela lacking very long setae overreaching setae in tufts	0		
	Chela bearing very long setae overreaching setae in tufts	1	3I	
**THIRD PEREOPOD**				
18	Propodus lacking specialized strong curved spines proximal to tufts of setae	0		
	Propodus bearing specialized strong curved spines proximal to tufts of setae	1	3J	
19	Fixed finger in chela rudimentary, shorter then dactyl	0	3J	
	Fixed finger developed, as long as dactyl	1	3K	
20	Chela lacking very long setae overreaching setae in tufts	0		
	Chela bearing very long setae overreaching setae in tufts	1	3J	
**FIFTH PEREOPOD**				
21	Propodus setose along both margins	0		
	Propodus setose along one margin only	1		
**UROPODAL EXOPOD**				
22	Outer spine absent	0		
	Outer spine present	1	4A	
23	Proximal segment not setose along outer margin	0		
	Proximal segment partly setose along outer margin	1		
	Proximal segment entirely setose along outer margin	2		
**MALE CLASPING ORGAN**				
24	Rudimentary	0	4B	
	Developed	1	4C	
25	Serrated bristles absent	0		
	Serrated bristles present, 1–7	1		
	Serrated bristles present, 8–13	2	4E	
26	Serrated bristles positioned in an unordered heap	0		
	Serrated bristles positioned in an ordered row	1	4E	
27	Tubercle absent	0	4B	
	Tubercle present	1	4C–E	
**PETASMA**				
28	Lobus armatus absent	0		
	Lobus armatus present	1	5A–E	
29	Lobus armatus rudimentary	0	5E	
	Lobus armatus developed	1	5A–D	
30	Lobus connectens and lobus terminalis not twisted	0	5A–E	
	Lobus connectens and lobus terminalis twisted	1	6D	
31	Lobus connectens absent	0	5B	
	Lobus connectens present	1	4A,C–E	
32	Lobus connectens rudimentary	0	5C	
	Lobus connectens developed	1	5A,D,E	
33	Lobus connectens entire	0	5A,C,D	
	Lobus connectens divided	1	5E	
	Lobus connectens with additional lobe at base	2	6C	
34	Lobus connectens not swan-shaped	0	6B,C	
	Lobus connectens swan-shaped	1	6E	
35	Lobus connectens without pillow at base	0	6C,D	
	Lobus connectens with pillow at base	1	6E	
36	Lobus inermis straight	0	5A,B,E	
	Lobus inermis curved	1	6A	
37	Lobus inermis narrow	0	5B	
	Lobus inermis inflated	1	6A	
38	Lobus terminalis rudimentary	0		
	Lobus terminalis developed	1	5A–D	
39	Lobus terminalis entire	0	5A–D	
	Lobus terminalis divided	1	6A	
40	Processus uncifer without terminal hook	0	5C, 6D	
	Processus uncifer with terminal hook	1	5A,B,D	
41	Processus ventralis absent	0	5C	
	Processus ventralis present	1	5A,B,D,E	
42	Processus ventralis rudimentary	0	5E	
	Processus ventralis developed	1	5A,B,D	
43	Processus ventralis entire	0	5A,B,D,E	
	Processus ventralis divided	1		
44	Processus ventralis elongate	0	5A,B,D	
	Processus ventralis triangle	1	5E	
45	Processus ventralis without hooks and sucks	0	5A–E	
	Processus ventralis with hooks and sucks	1	6B	
46	Processus ventralis without simple spines	0	5A,C,E	
	Processus ventralis with simple spines	1	5B,D	
47	Processus ventralis without stellate spines	0	5A,D,E	
	Processus ventralis with stellate spines	1	5B	
48	Processus ventralis with a single stellate spine	0		
	Processus ventralis with 4 or more stellate spines	1	5B	
49	Processus ventralis without apical lashes	0	5B–E	
	Processus ventralis with apical lashes	1	5A	
**PHOTOPHORES**				
50	The organ of Pesta absent		0	2F
	The organ of Pesta present		1	2E
51	Dermal photophores absent		0	
	Dermal photophores lens-less		1	7C–F
	Dermal photophores lens-bearing		2	7A,B
52	A total of 130–170 dermal photophores		0	
	A total of 190–210 dermal photophores		1	
	A total of 225 or more dermal photophores		2	
53	Dermal photophores arranged in a single lateral row on carapace		0	7B
	Dermal photophores arranged in 2 lateral rows on carapace		1	7A
54	Number of dermal photophores in the upper row on carapace fixed		0	
	Number of dermal photophores in the upper row on carapace not fixed		1	
55	Three or less dermal photophores in the upper row on carapace		0	
	Four or more dermal photophores in the upper row on carapace		1	7A,B
56	Dermal lens-less photophores arranged on scaphocerite positioned close to each other (distance between organs 4 times or less then diameter of organs)		0	7E
	Dermal lens-less photophores on scaphocerite much spaced from each other (distance between organs 5 times or more then diameter of organs)		1	7C
57	Number of dermal photophores on scaphocerite not fixed		0	
	Number of dermal photophores on scaphocerite fixed		1	
58	A total of 8 or more dermal photophores on scaphocerite		0	7C,E
	A total of 7 dermal photophores on scaphocerite		1	
	A total of 4–6 dermal photophores on scaphocerite		2	7G
	A total of 2–3 dermal photophores on scaphocerite		3	
59	Dermal photophores on scaphocerite large		0	7C
	Dermal photophores on scaphocerite small		1	7E
60	Dermal photophores on scaphocerite partly fused		0	7C
	Dermal photophores on scaphocerite separated from each other		1	7E
61	Dermal photophores arranged on scaphocerite in a single longitudinal row		0	7G,E
	Dermal photophores arranged on scaphocerite in 2 rows, longitudinal and oblique		1	7C
62	Dermal lens-less photophores arranged on uropodal exopod close to each other (distance between organs 4 times or less then diameter of organs)		0	7D
	Dermal lens-less photophores on uropodal exopod much spaced from each other (distance between organs 5 times or more then diameter of organs)		1	7F,H
63	Dermal photophores on uropodal exopod large		0	7D
	Dermal photophores on uropodal exopod small		1	7F,H
64	Dermal photophores on uropodal exopod separated from each other		0	7F
	Dermal photophores on uropodal exopod partly fused		1	7D
65	Dermal photophores on basal segment of uropodal exopod positioned closer to central axis		0	7D,H
	Dermal photophores on basal segment of uropodal exopod positioned closer to margin		1	7F
66	Number of dermal photophores on basal segment of uropodal exopod not fixed		0	
	Number of dermal photophores on basal segment of uropodal exopod fixed		1	
67	A total of 3 or more dermal photophores on basal segment of uropodal exopod		0	7F
	Two dermal photophores on basal segment of uropodal exopod		1	
	A single dermal photophore on basal segment of uropodal exopod		2	7H
68	Dermal photophores on distal segment of uropodal exopod positioned closer to central axis		0	7D,H
	Dermal photophores on distal segment of uropodal exopod positioned closer to margin		1	7F
69	Dermal photophores on distal segment of uropodal exopod arranged in a single row		0	7F,H
	Dermal photophores on distal segment of uropodal exopod arranged in 2 rows/triangle		1	7D
70	Number of dermal photophores on distal segment of uropodal exopod not fixed		0	
	Number of dermal photophores on distal segment of uropodal exopod fixed		1	
71	A total of 3 or more dermal photophores on distal segment of uropodal exopod		0	7F
	A single dermal photophore on distal segment of uropodal exopod		1	7H
	No dermal photophores on distal segment of uropodal exopod		2	

### 4. Phylogenetic Analysis, Outgroup, and Character Optimization

Data were handled and analyzed using a combination of a number of programs using maximum parsimony: Winclada/Nona, NDE (Nexus Data Editor), TNT, and Mesquite [20] – [22]. The trees on which the classificatory changes (e.g., erection of six new genera) and evolutionary consideration (e.g., zoogeography) are built were generated in TNT using the ‘traditional search’ options. The search parameters were set to the following: memory set to hold 1.000.000 trees; 1000 replicates with tree bisection-reconnection (TBR) branches swapping and saving 1000 trees per replicate; zero-length branches collapsed; suboptimal trees were set to be filtered out.

*Sicyonella antennata* (Sergestidae) was used as the outgroup. This species is clearly outside the *Sergia/Sergestes* species complex, and, following [23], there is some evidence that its morphology may be close to a common sergestid ancestor. It is common opinion (to be tested phylogenetically) that pereopods have been gradually reduced within Sergestidae possibly as an adaption to a pelagic life style. *Sicyonella antennata* (outgroup), which occurs in near-bottom water layers [23] – [24] where many groups of shrimps are known to have evolved [25], has, with other species in the genus, the least reduced pereopods within the family, e.g., with chelae on pereopod 1 and dactyli on pereopods 4 and 5. Species of this genus also have well-developed gills (branchiae) and no luminous organs.

**Figure 8:**
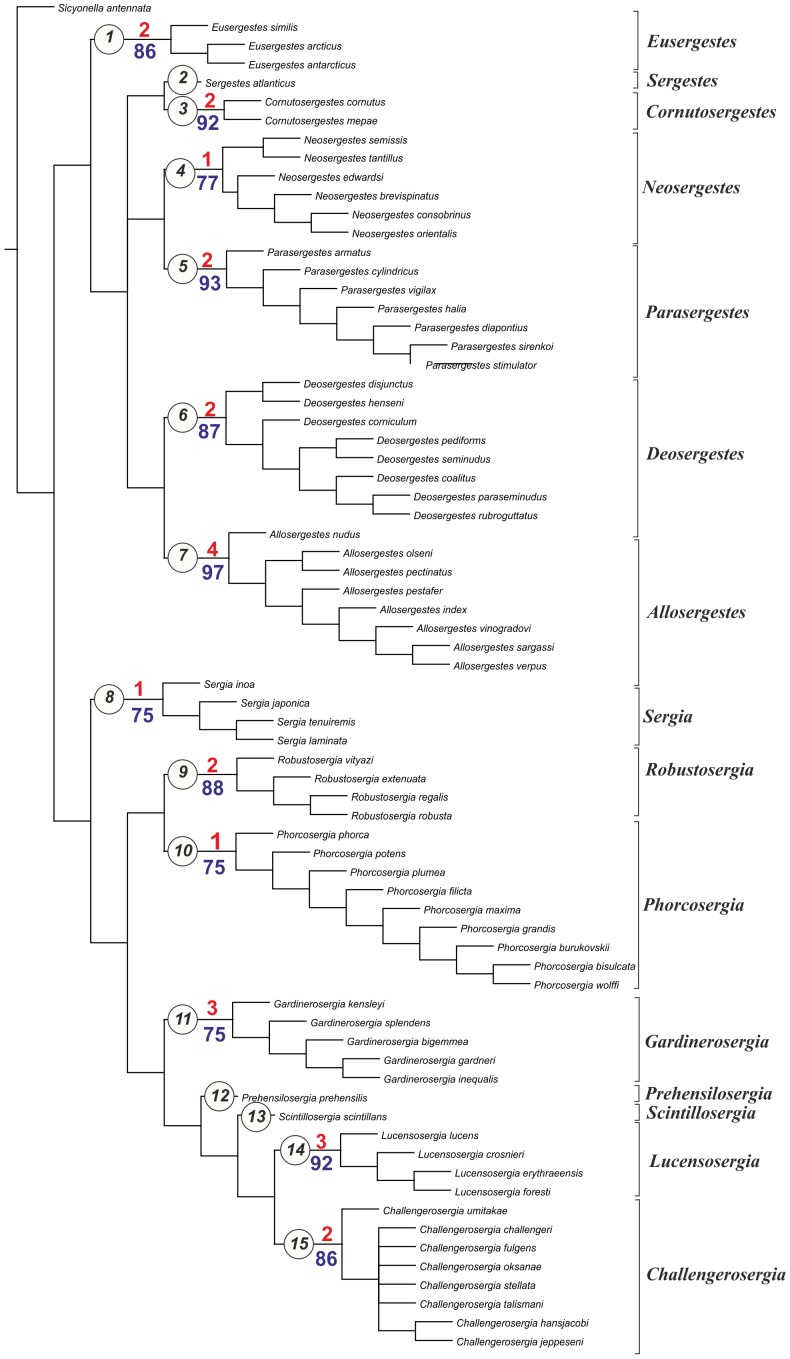
The strict consensus tree, principal clades (black, in circles) and their Bremer support (red) and Bootstrap support (blue).

Characters were mapped on trees using TNT's character mapping functions. Some of these characters (synapomorphies) are listed in Table 4 and shown on the strict consensus tree in Fig. 8. In general, we preferred character optimizations that favored primary homology (ACCTRAN).

**Table 4:** List of the clades with most important supporting characters shown divided into major character systems (non-sexual, sexual, and photophore synapomorphies).

Clade number	Taxon name (Fig. 8)	Bremer support	Bootstrap support	Supporting characters (marked with character number)			Species included
				Synapomorphies in general decapod anatomy	Sexual synapomorphies	Photophore synapomorphies	
1	*Eusergestes*	2	86	(10) Maxilliped III sexually dimorphic	(46) PETASMA: PV with simple spines		*antarcticus*
							*arcticus*
							*similis*
2	*Sergestes*	n/a	n/a		(29) PETASMA: LA rudimentary		*atlanticus*
					(33) PETASMA: LC divided		
					(44) PETASMA: PV triangle		
3	*Cornutosergestes*	2	**92**	(8) Reduced 2-segmented maxilliped	(32) PETASMA: LC rudimentary		*cornutus*
					(41) PETASMA: PV absent		*mepae*
4	*Neosergestes*	1	77	(23) Proximal segment of UP exopod entirely setose along outer margin	(39) PETASMA: LT divided		*brevispinatus*
							*consobrinus*
							*edwardsii*
							*orientalis*
							*semissis*
							*tantillus*
5	*Parasergestes*	2	**93**	(12) Dactyl of maxilliped III consist of 4 specialized subsegments	(32) PETASMA: LC rudimentary		*armatus*
				(13) Ischium of pereopod I with strong movable spines			*cylindricus*
							*diapontius*
							*halia*
							*sirenkoi*
							*stimulator*
							*vigilax*
6	*Deosergestes*	2	87	(17) Chela of pereopod II with very long setae overreaching setae in tufts	(49) PETASMA: PV with apical lashes		*coalitus*
							*corniculum*
							*disjunctus*
							*henseni*
							*paraseminudus*
							*pediformis*
							*rubroguttatus*
							*seminudus*
7	*Allosergestes*	**4**	**97**	(1) Frontal margin of rostrum vertical	(31) PETASMA: LC absent		*index*
				(16) Chela of pereopod II with rudimentary fixed finger	(46) PETASMA: PV with simple spines		*nudus*
							*oleseni*
							*pectinatus*
							*pestafer*
							*sargassi*
							*verpus*
							*vinogradovi*
8	*Sergia*	1	75	(0) Membranous integument			*inoa*
							*japonica*
							*laminata*
							*tenuiremis*
9	*Robustosergia*	2	88		(30) PETASMA: LC and LT twisted		*extenuata*
					(35) PETASMA: LC with pillow at base		*regalis*
							*robusta*
							*vityazi*
10	*Phorcosergia*	1	75		(33) PETASMA: LC divided	(59–61) Photophores on scaphocerite large, partly fused, and arranged in 2 rows.	*bisulcata*
						(63–64) Photophores on uropodal exopod large, partly fused	*burukovskii*
						(69) Photophores on distal segment of uropodal exopod arranged in 2 rows/triangle	*filicta*
							*grandis*
							*maxima*
							*phorca*
							*plumea*
							*potens*
							*wolffi*
11	*Gardinerosergia*	**3**	75	(5) Ocular papilla developed		(65) Photophores on basal segment of uropodal exopod positioned closer to margin	*bigemmea*
						(68) Photophores on distal segment of uropodal exopod positioned closer to margin	*gardineri*
							*inequalis*
							*kensleyi*
							*splendens*
12	*Prehensilosergia*	n/a	n/a		(33) PETASMA: LC divided	(63) 10–15 organs on scaphocerite, No not fixed	*prehensilis*
						(65) 4–8 organs on proximal segment of uropodal exopod number not fixed	
						(68) 3–5 organs on distal segment of uropodal exopod, number not fixed	
13	*Scintillosergia*	n/a	n/a		(38) PETASMA: LT rudimentary		*scintillans*
					(46) PETASMA: PV absent		
14	*Lucensosergia*	**3**	**92**		(32) PETASMA: LC rudimentary	(55) Reduced number of photophores in the upper row on carapace	*crosnieri*
					(45) PETASMA: PV with hooks and sucks		*erythraeensis*
							*foresti*
							*lucens*
15	*Challengerosergia*	2	86		(25–26) Male clasping organ: 8 or more strong serrated stout spines positioned in an ordered row		*challengeri*
					(36) PETASMA: LI curved		*fulgens*
							*hansjabobi*
							*jeppeseni*
							*oksanae*
							*stellata*
							*talismani*
							*umitakae*

Bremer support ≥3 and Bootstrap support ≥90 are in bold.

Bremer support was calculated with the use of the program TNT, algorithm TBR, with the following settings: for all suboptimal trees, trees searched with a score of up to 2–15 worse than best (in 2–15 searches, each one worse than previous), saving up to 1000 trees.

Bootstrap support was calculated with the use of the program TNT, standard (sample with replacement), with the following settings: 10000 replicates, traditional search, groups collapse below 75.

### 5. Nomenclatural Acts

The electronic edition of this article conforms to the requirements of the amended International Code of Zoological Nomenclature, and hence the new names contained herein are available under that Code from the electronic edition of this article. This published work and the nomenclatural acts it contains have been registered in ZooBank, the online registration system for the ICZN. The ZooBank LSIDs (Life Science Identifiers) can be resolved and the associated information viewed through any standard web browser by appending the LSID to the prefix “http://zoobank.org/”. The LSID for this publication is: urn:lsid:zoobank.org:pub: 1573AF28-5DD4-47ED-AACD-2C2DDCB47E02. The electronic edition of this work was published in a journal with an ISSN, and has been archived and is available from the following digital repositories: PubMed Central, LOCKSS.

## Results and Discussion

### 1. The Clades

All equally weighted parsimony analyses of the complete data set in TNT (traditional search) resulted in 8 equally short trees. The strict consensus tree is rather resolved, especially the deeper nodes, so classificatory conclusions have been based on this tree (Fig. 8). Further we consider only the clades corresponding to the genera, supported by synapomorphies, and having Bootstrap support 75 or more. There is no support for *Sergia* and *Sergestes* genera as they have been defined until now. *Sergia* and *Sergestes* are therefore both redefined with a less inclusive content as are a number of smaller genera, some new and some proposed by [18].

All characters fall into one of three groups: (1) general decapod anatomy, (2) male sexual characters (clasping organ, petasma), and (3) photophore patterns. Table 4 shows that the clades are supported by synapomorphies relating to both sexual and non-sexual characters in different proportions for various clades.

***Clade 1*** is supported by 2 synapomorphies related to general anatomy and sexual structures. The clade corresponds to the former “*Sergestes arcticus*” species group (erected by Yaldwyn [5]; see [8]) and *Eusergestes* [18]. The clade includes three species. Our phylogenetic analysis supports the genus *Eusergestes* erected by Judkins and Kensley [18].

***Clade 2*** is supported by 3 synapomorphies related to sexual structures and includes the only species *Sergestes atlanticus.* Our phylogenetic analysis supports generic status of this group (isolated species in [8]).

***Clade*** ***3*** is supported by one non-sexual synapomorphy and 2 synapomorphies related to sexual structures and includes the former “*Sergestes cornutus*” species group (erected by Vereshchaka [8]). Bootstrap support of this group is remarkably high (92). The clade includes two species. On the basis of phylogenetic analysis we raise the status of this species group to generic level. In order to maintain the continuity of the systematics of *Sergestes* and *Sergia* we name the new genus *Cornutosergestes.*

***Clade 4*** is supported by one non-sexual synapomorphy and one synapomorphy related to sexual structures and corresponds to the former “*Sergestes edwardsi*” species group [5], [8] and the genus *Neosergestes* [18]. The clade includes six species. Our phylogenetic analysis supports the genus *Neosergestes* which was erected by Judkins and Kensley [18].

***Clade 5*** is supported by two general morphology synapomorphies and one synapomorphy related to sexual structures. Bootstrap support of this group is very high (93). The clade includes seven species and corresponds to the former “*Sergestes vigilax*” species group [5], [8] and the genus *Parasergestes* [18]. Our phylogenetic analysis supports the genus *Parasergestes* erected by Judkins and Kensley [18].

***Clade 6*** is supported by one general morphology synapomorphy and one synapomorphy related to sexual structures and corresponds to the former “*Sergestes corniculum*” species group [5], [8] and the genus *Deosergestes* [18]. The clade includes 8 species. Our phylogenetic analysis supports the erection of the genus *Deosergestes* by Judkins and Kensley [18].

***Clade 7*** is supported by 2 general morphology synapomorphies and 2 synapomorphies related to sexual structures and corresponds to the former “*Sergestes sargassi*” species group [5], [8] and the genus *Allosergestes* [18]. This is the best supported clade (Bootstrap support 97, Bremer support 4). The clade includes eight species. Our phylogenetic analysis supports the genus *Allosergestes* which was erected by Judkins and Kensley [18].

***Clade 8*** is supported by one synapomorphy related to general morphology. This clade includes 4 species belonging to the former “*Sergia japonica*” species group along with the isolated species *Sergia tenuiremis* and *Sergia inoa* [7]. We recognised this clade as a separate genus *Sergia*.

***Clade 9*** is supported by 2 synapomorphies related to sexual structures and corresponds to the former “*Sergia robusta*” species group [7]. The clade includes four species and is recognised as a new genus *Robustosergia*.

***Clade 10*** is supported by one sexual synapomorphy and 6 synapomorphies related to photophore characters. The clade corresponds to the former “*Sergestes phorca*” species groups [7], includes 9 species, and is recognised as a new genus *Phorcosergia*.

***Clade 11*** is supported by one synapomorphy related to general morphology and 2 synapomorphies related to photophore characters. The Bremer support is significant for this clade. The clade corresponds to the former “*Sergia gardineri*” species group [7], includes five species, and is recognised as a new genus *Gardinerosergia*.

***Clade 12*** is supported by one sexual synapomorphy and 3 photophore-related synapomorphies and includes a single species, *Sergia prehensilis.* Vereshchaka [8] placed *Sergia prehensilis* and *Sergia scintillans* together in a “*Sergia prehensilis*” species group. Our phylogenetic analysis splits this group into two parts with *Sergia prehensilis* placed in a separate monotypic genus. Clade 12 is recognised as a new genus *Prehensilosergia*.

***Clade 13*** is supported by 2 synapomorphies related to sexual structures and includes the single species *Sergia scintillans,* which is recognised now as a new monotypic genus *Scintillosergia*.

***Clade 14*** is supported by 2 sexual synapomorphies and one photophore-related synapomorphy and corresponds to the former “*Sergia lucens*” species group [7]. The clade includes four species and is now recognised as a new genus *Lucensosergia*.

***Clade 15*** is supported by 3 synapomorphies related to sexual structures and corresponds to the former “*Sergia challengeri*” species group [7]. The clade includes eight species and is now recognised as a new genus *Challengerosergia*.

### 2. New Genera and their Diagnoses

As a result of the phylogenetic analysis, eight new genera (Fig. 8) are erected. Thus, the former genera *Sergestes* and *Sergia* now include 15 genera and 71 species (7 genera and 35 species of the former genus *Sergestes* and 8 genera and 36 species of the former genus *Sergia*). Below are emended diagnoses for all recognized genera listed in alphabetical order.

***Allosergestes*** **Judkins, Kensley, 2008**

**Diagnosis**: Integument firm, frontal margin of rostrum vertical, hepatic protrusion forming tooth. First segment of A I not elongate, <1.5 times as long as 3d segment; distoventral end of 3rd segment in males without process; distal tooth of scaphocerite reaching or overreaching end of blade; maxilliped I developed, 3-4-segmented, maxilliped III> 2.0 times as long as Cp, not dimorphic sexually, dactyl subdivided into 5-6 specialized subsegments; pereopods I–II ischia without strong movable spines and distally curved tooth; pereopod II with merus lacking protrusion and chela bearing fixed finger shorter than dactyl, without long setae; chela of pereopod III with strong curved spines and long setae on propodus, fixed finger shorter than dactyl; pereopods IV–V present, 6-segmented; pereopod V setose along both margins; uropodal exopod without outer spine, proximal segment partly setose along outer margin. Male clasping organ: if developed, without serrated bristles, tubercle present. Petasma: LA well-developed, LC absent, LI well-developed, straight, slender, LT well-developed, PU present, with hook, PV well-developed, elongate, entire, with simple or stellate spines. Photophores: organ of Pesta present, dermal organs absent. Arthrobranchs: posterior lobe on somite XII lamellar, anterior lobe on somite XIII dendritic.

**Type species**: By designation of Judkins, Kensley (2008) [18], *Allosergestes sargassi* Ortmann, 1893 [26].

**Type locality**: Florida Current, Sargasso Sea.

**Etymology**: from the Greek ‘αλλ*ο*σ” meaning other plus the root *‘sergestes’.*)

**Species included**: *Allosergestes index* (Burkenroad, 1940) [27], *Allosergestes nudus* (Illig, 1914) [28], *Allosergestes oleseni* (Vereshchaka, 2009) [8], *Allosergestes pectinatus* (Sund, 1920) [29], *Allosergestes pestafer* (Burkenroad, 1937) [30], *Allosergestes sargassi* (Ortmann, 1893) [26], *Allosergestes verpus* (Burkenroad, 1940) [27], and *Allosergestes vinogradovi* (Vereshchaka, 2009) [8].

*Challengerosergia* **gen.n.**

urn:lsid:zoobank.org:act:D3570D73-67D1-4AD6-98AB-AB064FFF346B

**Diagnosis**: Integument firm, frontal margin of rostrum oblique, no supraorbital tooth, hepatic protrusion forming tooth. First segment of A I elongate, ≥1.5 times as long as 3d segment; distal tooth of scaphocerite reaching or overreaching end of blade; maxilliped I developed, 3-4-segmented, maxilliped III<2.0 times as long as Cp, not dimorphic sexually, dactyl not subdivided into specialized subsegments; pereopods I–II ischia without strong movable spines and distally curved tooth; pereopod II with merus lacking protrusion and chela bearing equal fingers, without long setae; chela of pereopod III without strong curved spines and long setae on propodus, fingers subequal; pereopods IV–V present, 6-segmented; pereopod V setose along both margins; uropodal exopod with outer spine, proximal segment not setose along outer margin. Male clasping organ: if developed, with 8–13 serrated bristles in an ordered row and tubercle present. Petasma: LA rudimentary, LC well-developed, entire, without pillow at base, twisted with LT, LI well-developed, curved, inflated, LT well-developed, PU present, with hook, PV well-developed, entire, elongate, without hooks, suckers, spines, or apical lashes. Photophores: dermal, lens-bearing, small, position fixed; arranged in 1 row on carapace (4–6 organs), in 1 row on scaphocerite (4–6 organs) and uropodal exopod (1–2 organs on basal segment and 1 organ on distal segment); positioned close to central axis of scaphocerite and uropodal exopod. Arthrobranchs: both posterior lobe on somite XII and anterior lobe on somite XIII dendritic.

**Type species**: by present designation, *Challengerosergia challengeri* (Hansen, 1903) [4]

**Type locality:** Western Pacific off Matuku, Fiji Islands, 19^o^9'35″S, 179^o^41'50″E.

**Etymology**: after type species *P. challengeri* (the name of the famous British Challenger Expedition) plus the root *‘sergia’*.

**Species included**: Challengerosergia challengeri (Hansen, 1903) [4], Challengerosergia fulgens (Hansen, 1919) [13], Challengerosergia hansjacobi (Vereshchaka, 1994) [19], Challengerosergia jeppeseni (Vereshchaka, 2000) [7], Challengerosergia oksanae (Vereshchaka, 2000) [7], Challengerosergia stellata (Burkenroad, 1940) [27], Challengerosergia talismani (Barnard, 1946) [31], Challengerosergia umitakae (Hashizume, Omori, 1995) [32].

*Cornutosergestes* **gen. n.**

urn:lsid:zoobank.org:act:9E3D59AA-5A73-468F-813C-54652CFAB8A6

**Diagnosis**: Integument firm, frontal margin of rostrum oblique, supraorbital tooth present, hepatic protrusion forming tooth. First segment of A I not elongate, <1.5 times as long as 3d segment; distoventral end of 3rd segment in males without process; distal tooth of scaphocerite reaching or overreaching end of blade; maxilliped I reduced, 2-segmented, maxilliped III<2.0 times as long as Cp, not dimorphic sexually, dactyl subdivided into 4–6 specialized subsegments; pereopods I–II ischia without strong movable spines and distally curved tooth; pereopod II with merus lacking protrusion and chela bearing equal fingers, without long setae; chela of pereopod III without strong curved spines and without long setae on propodus, fixed finger subequal to dactyl; pereopods IV–V present, 6-segmented; pereopod V setose along one margin; uropodal exopod with outer spine, proximal segment not setose along outer margin. Male clasping organ: developed, without serrated bristles, tubercle present. Petasma: LA developed, LC rudimentary, entire, without pillow at base, twisted with LT, LI slender, LT well-developed, entire, PU present, without hook, PV absent. Photophores: organ of Pesta present, dermal organs absent. Arthrobranchs: posterior lobe on somite XII lamellar, anterior lobe on somite XIII dendritic.

**Type species**: by present designation, *Cornutosergestes cornutus* Krøyer, 1855 [33]

**Type locality:** Type locality: Central Atlantic, 10°N, 30°W (information from type's label)

**Etymology**: after type species *C. cornutus* (from the Latin ‘*cornutus”* meaning horned, probably an allusion to its elongate rostrum, plus the root *‘sergestes’*)

**Species included**: *Cornutosergestes cornutus* (Krøyer, 1855) [33] and *Cornutosergestes mepae* (Vereshchaka, 2009) [8].

*Deosergestes* **Judkins, Kensley, 2008**

**Diagnosis**: Integument firm, frontal margin of rostrum oblique, hepatic protrusion forming tooth. First segment of A I not elongate, <1.5 times as long as 3d segment; distoventral end of 3rd segment in males without process; distal tooth of scaphocerite reaching or overreaching end of blade; maxilliped I developed, 3-4-segmented, maxilliped III<2.0 times as long as Cp, not dimorphic sexually, dactyl subdivided into 6–7 specialized subsegments; pereopods I–II ischia without strong movable spines and distally curved tooth; pereopod II with merus lacking protrusion and chela bearing equal fingers, with long setae; chela of pereopod III with strong curved spines and long setae on propodus, fixed finger shorter than dactyl; pereopods IV–V present, 6-segmented; pereopod V setose along both margins; uropodal exopod without outer spine, proximal segment partly setose along outer margin. Male clasping organ: developed, without serrated bristles, tubercle present. Petasma: LA well-developed, LC well-developed, entire, without pillow at base, twisted with LT, LI well-developed, straight, slender, LT well-developed, entire, PU present, with hook, PV well-developed, elongate, with apical lashes. Photophores: organ of Pesta present, dermal organs absent. Arthrobranchs: both posterior lobe on somite XII and anterior lobe on somite XIII dendritic.

**Type species**: by present designation, *Deosergestes corniculum* Krøyer, 1855 [33]. Judkins, Kensley (2008) [18] offered *Sergestes curvatus* Crosnier, Forest, 1973 [34], but this species was synonymized with *Sergestes corniculum* Krøyer, 1855 by Vereshchaka (2009) [8].

**Type locality**: Tropical Atlantic, ca. 41/2°N, 211/2°W, coll. Hr. Fries [information from Danish introduction in Krøyer 1855[33]].

**Etymology**: from the Greek *‘δεω’* meaning to tie up, plus the root *‘sergestes’.*

**Species included**: *Deosergestes coalitus* (Burkenroad, 1940) [27], *Deosergestes corniculum* (Krøyer, 1855[33]), *Deosergestes disjunctus* (Burkenroad, 1940) [27], *Deosergestes henseni* (Ortmann, 1893) [26], *Deosergestes paraseminudus* (Crosnier, Forest, 1973) [34], *Deosergestes pediformis* (Crosnier, Forest, 1973) [34], *Deosergestes rubroguttatus* (Wood-Mason, 1891 *in* Wood-Mason, Alcock 1891 [35]), and *Deosergestes seminudus* (Hansen, 1919) [13].

*Eusergestes* **Judkins, Kensley, 2008**

**Diagnosis**: Integument firm, frontal margin of rostrum oblique, supraorbital tooth present, hepatic protrusion forming tooth. First segment of A I elongate,>1.5 times as long as 3d segment; distoventral end of 3rd segment in males without process; distal tooth of scaphocerite reaching or overreaching end of blade; maxilliped I developed, 3-4-segmented, maxilliped III <2.0 times as long as Cp, dimorphic sexually, dactyl subdivided into 6 specialized subsegments; pereopods I–II ischia without strong movable spines and distally curved tooth; pereopod II with merus lacking protrusion and chela bearing equal fingers, without long setae; chela of pereopod III without strong curved spines and without long setae on propodus, fixed finger subequal to dactyl; pereopods IV–V present, 6-segmented; pereopod V setose along one margin; uropodal exopod with outer spine, proximal segment not setose along outer margin. Male clasping organ: developed, without serrated bristles, tubercle present. Petasma: LA well-developed, LC well-developed, entire, without pillow at base, twisted with LT, LI absent, LT well-developed, entire, PU present, with hook, PV well-developed, entire, elongate, with simple spines. Photophores: organ of Pesta present, dermal organs absent. Arthrobranchs: posterior lobe on somite XII lamellar, anterior lobe on somite XIII dendritic.

**Type species**: By designation of Judkins, Kensley (2008) [18], *Eusergestes arcticus* Krøyer, 1855 [33].

**Type locality:** Atlantic Ocean, off Western Greenland (the only locality information given in Krøyer, 1855 [33])

**Etymology**: from the Greek ‘ευ-” meaning true plus the root *‘sergestes’.*)

**Species included**: *Eusergestes antarcticus* (Vereshchaka, 2009) [8], *Eusergestes arcticus* (Krøyer, 1855) [33], and *Eusergestes similis* (Hansen, 1903) [4].

*Gardinerosergia* **gen.n.**

urn:lsid:zoobank.org:act:887F6724-DB45-4030-AE6A-D4DB0A684572

**Diagnosis**: Integument firm, frontal margin of rostrum oblique, no supraorbital or hepatic teeth, hepatic protrusion forming barb. First segment of A I elongate, ≥1.5 times as long as 3d segment; distoventral end of 3rd segment in males without process; distal tooth of scaphocerite reaching or overreaching end of blade; maxilliped I developed, 3-4-segmented, maxilliped III<2.0 times as long as Cp, not dimorphic sexually, dactyl not subdivided into specialized subsegments; pereopods I–II ischia without strong movable spines and distally curved tooth; pereopod II with merus lacking protrusion and chela bearing equal fingers, without long setae; chela of pereopod III without strong curved spines and long setae on propodus, fingers subequal; pereopods IV–V present, 6-segmented; pereopod V setose along both margins; uropodal exopod with outer spine, proximal segment not setose along outer margin. Male clasping organ: developed, with 1–7 serrated bristles in an unordered heap, tubercle present. Petasma: LA well-developed, LC well-developed, without pillow at base, twisted with LT, LI well-developed, straight, slender, LT well-developed, entire, PU present, with hook, PV well-developed, entire, elongate, without hooks, suckers, spines, or apical lashes. Photophores: dermal, as opaque spots, small, not fused, position not fixed; arranged in 2 rows on carapace, in 1 row on scaphocerite and uropodal exopod; positioned close to central axis of scaphocerite and close to margin of uropodal exopod. Arthrobranchs: both posterior lobe on somite XII and anterior lobe on somite XIII dendritic.

**Type species**: by present designation, *Gardinerosergia gardineri* (Kemp, 1913) [36]

**Type locality**: Western Indian Ocean: S by E of Farquhar, 10°27′S, 51°17′E, 27 Sep. 1905 (3 different samples: 2 young, badly damaged; 3 males, 3 females, 15–24 mm; 1 female, 20 mm); NE of Madagascar, between Providence and Alphonse Islands, 8°16′S, 51°26′E, 6 Oct. 1905 (1 male, 17 mm); 5 miles off Desroches Atoll (1 male, 17 mm).

**Etymology**: after type species *G. gardineri* (the species named after Mr. J. Stanley Gardiner, who collected the type species).

**Species included**: Gardinerosergia bigemmea (Burkenroad, 1940) [27], Gardinerosergia gardineri (Kemp, 1913) [36], Gardinerosergia inequalis (Burkenroad, 1940) [27], Gardinerosergia kensleyi (Vereshchaka, 2000) [7], and Gardinerosergia splendens (Sund, 1920) [29].

*Lucensosergia* **gen.n.**

urn:lsid:zoobank.org:act:2DC34293-FA28-4AF5-928B-499DF445D372

**Diagnosis**: Integument firm, frontal margin of rostrum oblique, no supraorbital tooth, hepatic protrusion forming tooth. First segment of A I elongate, ≥1.5 times as long as 3d segment; distoventral end of 3rd segment in males without process; distal tooth of scaphocerite reaching or overreaching end of blade; maxilliped I developed, 3-4-segmented, maxilliped III<2.0 times as long as Cp, not dimorphic sexually, dactyl not subdivided into specialized subsegments; pereopods I–II ischia without strong movable spines and distally curved tooth; pereopod II with merus lacking protrusion and chela bearing equal fingers, without long setae; chela of pereopod III without strong curved spines and long setae on propodus, fingers subequal; pereopods IV–V present, 6-segmented; pereopod V setose along both margins; uropodal exopod with outer spine, proximal segment not setose along outer margin. Male clasping organ: developed, with serrated bristles in an unordered heap, tubercle present. Petasma: LA absent or rudimentary, LC rudimentary, entire, without pillow at base, twisted with LT, LI well-developed, straight, slender, LT well-developed, entire, PU present, with hook, PV well-developed, entire, elongate, with hooks and suckers. Photophores: dermal, lens-bearing, small, position fixed; arranged in 1 row on carapace (2–3 organs), in 1 row on scaphocerite (2–3 organs) and uropodal exopod (1 organ on basal segment and 0–1 organ on distal segment); positioned close to central axis of scaphocerite and uropodal exopod. Arthrobranchs: both posterior lobe on somite XII and anterior lobe on somite XIII dendritic.

**Type species**: by present designation, *Lucensosergia lucens* (Hansen, 1922) [15]

**Type locality:** Type locality: Suruga Bay, Japan

**Etymology**: after type species *L. lucens* (from the Latin ‘*lucens”*  =  “*lucentis”* meaning lighting, probably an allusion to the shrimp's numerous phosphorescent photophores, plus the root *‘sergia’*).

**Species included**: *Lucensosergia crosnieri* (Vereshchaka, 2000) [7], *Lucensosergia erythraeensis* (Iwasaki, Couwelaar, 2001) [37], *Lucensosergia foresti* (Kensley, Judkins, 2008) [18], and *Lucensosergia lucens* (Hansen, 1922) [15].

*Neosergestes* **Judkins, Kensley, 2008**

**Diagnosis**: Integument firm, frontal margin of rostrum oblique, supraorbital tooth absent, hepatic protrusion forming tooth. First segment of A I not elongate, <1.5 times as long as 3d segment; distoventral end of 3rd segment in males without process; distal tooth of scaphocerite reaching or overreaching end of blade; maxilliped I developed, 3-4-segmented, maxilliped III>2.0 times as long as Cp, not dimorphic sexually, dactyl subdivided into 6 specialized subsegments; pereopods I–II ischia with strong movable spines and distally curved tooth; pereopod II with merus having protrusion and chela bearing equal fingers, without long setae; chela of pereopod III without strong curved spines and without long setae on propodus, fixed finger subequal to dactyl; pereopods IV–V present, 6-segmented; pereopod V setose along one margin; uropodal exopod without outer spine, proximal segment entirely setose along outer margin. Male clasping organ: developed, without serrated bristles, tubercle present. Petasma: LA well-developed, LC well-developed, without pillow at base, twisted with LT, LI well-developed, straight, inflated, LT well-developed, divided, PU present, without hook, PV rudimentary, entire, elongate, without hooks, suckers, spines, or apical lashes. Photophores: organ of Pesta present, dermal organs absent. Arthrobranchs: both posterior lobe on somite XII and anterior lobe on somite XIII dendritic.

**Type species**: By designation of Judkins, Kensley (2008) [18], *Neosergestes edwardsii* Krøyer, 1855 [33].

**Type locality:** North Atlantic, 10°22′ N, 21°16′W.

**Etymology**: from the Greek ‘νεοσ’ meaning new plus the root *‘sergestes’.*)

**Species included**: *Neosergestes brevispinatus* (Judkins, 1978) [38], *Neosergestes consobrinus* (Milne, 1968) [39], *Neosergestes edwardsi* (Krøyer, 1855) [33], *Neosergestes orientalis* (Hansen, 1919) [13], *Neosergestes semissis* (Burkenroad, 1940) [27], and *Neosergestes tantillus* (Burkenroad, 1940) [27].

*Parasergestes* **Judkins, Kensley, 2008**

**Diagnosis**: Integument firm, frontal margin of rostrum oblique, supraorbital tooth absent, hepatic protrusion forming tooth. First segment of A I not elongate, <1.5 times as long as 3d segment; distoventral end of 3rd segment in males without process; distal tooth of scaphocerite reaching or overreaching end of blade; maxilliped I developed, 3-4-segmented, maxilliped III>2.0 times as long as Cp, not dimorphic sexually, dactyl subdivided into 4 specialized subsegments; pereopods I–II ischia with strong movable spines and distally curved tooth; pereopod II with merus having protrusion and chela bearing equal fingers, without long setae; chela of pereopod III without strong curved spines and without long setae on propodus, fixed finger subequal to dactyl; pereopods IV–V present, 6-segmented; pereopod V setose along one margin; uropodal exopod without outer spine, proximal segment partly setose along outer margin. Male clasping organ: developed, without serrated bristles, tubercle present. Petasma: LA well-developed, LC rudimentary, entire, without pillow at base, twisted with LT, LI well-developed, straight, inflated, LT well-developed, entire, PU present, without hook, PV rudimentary, elongate, entire, without hooks, suckers, spines, or apical lashes. Photophores: organ of Pesta present, dermal organs absent. Arthrobranchs: posterior lobe on somite XII lamellar, anterior lobe on somite XIII dendritic.

**Type species**: By designation of Judkins, Kensley (2008) [18], *Parasergestes armatus* Krøyer, 1855 [33].

**Type locality**: Equatorial Atlantic, 4°30′ N, 21°30′W.

**Etymology**: from the Greek ‘παρα-” meaning over or beside plus the root *‘sergestes’.*)

**Species included**: *Parasergestes armatus* (Krøyer, 1855) [33], *Parasergestes cylindricus* (Vereshchaka, 2009) [8], *Parasergestes diapontius* (Bate, 1881) [40], *Parasergestes halia* (Faxon, 1893) [41], *Parasergestes sirenkoi* (Vereshchaka, 2009) [8], *Parasergestes stimulator* (Burkenroad, 1940) [27], and *Parasergestes vigilax* (Stimpson, 1860) [10].

*Phorcosergia* **gen.n.**

urn:lsid:zoobank.org:act:A317C643-40A0-4941-A417-089931E69313

**Diagnosis**: Integument firm, frontal margin of rostrum oblique, no supraorbital or hepatic teeth, hepatic protrusion forming barb. First segment of A I elongate, ≥1.5 times as long as 3d segment; distoventral end of 3rd segment in males without process; distal tooth of scaphocerite not reaching end of blade; maxilliped I developed, 3-4-segmented, maxilliped III<2.0 times as long as Cp, not dimorphic sexually, dactyl not subdivided into specialized subsegments; pereopods I-II ischia without strong movable spines and distally curved tooth; pereopod II with merus lacking protrusion and chela bearing equal fingers, without long setae; chela of pereopod III without strong curved spines and long setae on propodus, fingers subequal; pereopods IV–V present, 6-segmented; pereopod V setose along both margins; uropodal exopod with outer spine, proximal segment not setose along outer margin. Male clasping organ: developed, without serrated bristles, tubercle present. Petasma: LA well-developed, LC well-developed, swan-shaped, without pillow at base, not twisted with LT, LI well-developed, straight, slender, LT well-developed, PU present, with hook, PV well-developed, entire, elongate, without hooks, suckers, spines, or apical lashes. Photophores: dermal, as opaque spots, large, partly fused, position not fixed; arranged in 2 rows on carapace, scaphocerite, and on distal segment of uropodal exopod; positioned close to central axis of scaphocerite and uropodal exopod. Arthrobranchs: both posterior lobe on somite XII and anterior lobe on somite XIII dendritic.

**Type species**: by present designation, *Phorcosergia phorca* (Faxon, 1893) [41].

**Type locality**: Eastern Pacific Ocean: Gulf of Panama; Galapagos; and Gulf of California (see details in [41]).

**Etymology**: after type species *P. phorca* (probably from the Latin ‘*forca”* meaning pitfall, snare, trap; plus the root *‘sergia’*)

**Species included:** *Phorcosergia bisulcata* (Wood-Mason in [35]), *Phorcosergia burukovskii* Vereshchaka, 2000 [7], *Phorcosergia filicta* (Burkenroad, 1940) [27], *Phorcosergia grandis* (Sund, 1920) [29], *Phorcosergia maxima* (Burkenroad, 1940) [27], *Phorcosergia phorca* (Faxon, 1893) [41], *Phorcosergia plumea* (Illig, 1927) [42], *Phorcosergia potens* (Burkenroad, 1940) [27], and *Phorcosergia wolffi* Vereshchaka, 1994 [19].

*Prehensilosergia* **gen.n.**

urn:lsid:zoobank.org:act:95104E59-08E6-4BED-B9CE-DD8028C0979F

**Diagnosis**: Integument firm, frontal margin of rostrum oblique, no supraorbital or hepatic teeth, hepatic protrusion forming barb. First segment of A I elongate, ≥1.5 times as long as 3d segment; distoventral end of 3rd segment in males without process; distal tooth of scaphocerite reaching or overreaching end of blade; maxilliped I developed, 3-4-segmented, maxilliped III<2.0 times as long as Cp, not dimorphic sexually, dactyl not subdivided into specialized subsegments; pereopods I–II ischia without strong movable spines and distally curved tooth; pereopod II with merus lacking protrusion and chela bearing equal fingers, without long setae; chela of pereopod III without strong curved spines and long setae on propodus, fingers subequal; pereopods IV–V present, 6-segmented; pereopod V setose along both margins; uropodal exopod with outer spine, proximal segment not setose along outer margin. Male clasping organ: developed, with 1–7 serrated bristles in an unordered heap, tubercle present. Petasma: LA well-developed, LC well-developed, divided, without pillow at base, twisted with LT, LI well-developed, straight, slender, LT well-developed, entire, PU present, with hook, PV well-developed, entire, elongate, without hooks, suckers, spines, or apical lashes. Photophores: dermal, lens-bearing, small, position not fixed; arranged in 2 rows on carapace, in 1 row on scaphocerite and uropodal exopod; positioned close to central axis of scaphocerite and uropodal exopod. Arthrobranchs: both posterior lobe on somite XII and anterior lobe on somite XIII dendritic.

**Type species**: by monotypy, *Prehensilosergia prehensilis* (Bate, 1881) [40].

**Type locality:** Western Pacific off Japan, 34^o^58′ N, 139^o^ 29′ E.

**Etymology**: after type species *P. prehensilis* (from the Latin ‘prehensilis*”* meaning prehensile, an allusion to heavily armed catching appendages, plus the root *‘sergia’*)

**Species included**: *Prehensilosergia prehensilis* (Bate, 1881) [40].

*Robustosergia* **gen.n.**

urn:lsid:zoobank.org:act:904D69CF-57C0-474B-95EB-3F871C7A95A5

**Diagnosis**: Integument firm, frontal margin of rostrum oblique, no supraorbital or hepatic teeth, hepatic protrusion forming barb. First segment of A I elongate, ≥1.5 times as long as 3d segment; distoventral end of 3rd segment in males without process; distal tooth of scaphocerite not reaching end of blade; maxilliped I developed, 3-4-segmented, maxilliped III<2.0 times as long as Cp, not dimorphic sexually, dactyl not subdivided into specialized subsegments; pereopods I–II ischia without strong movable spines and distally curved tooth; pereopod II with merus lacking protrusion and chela bearing equal fingers, without long setae; chela of pereopod III without strong curved spines and long setae on propodus, fingers subequal; pereopods IV–V present, 6-segmented; pereopod V setose along both margins; uropodal exopod with outer spine, proximal segment not setose along outer margin. Male clasping organ: developed, without serrated bristles, tubercle present. Petasma: LA well-developed, LC well-developed, entire, swan-shaped, with pillow at base, twisted with LT, LI well-developed, straight, slender, LT well-developed, entire, PU present, with hook, PV well-developed, entire, elongate, without hooks, suckers, spines, or apical lashes. Photophores: dermal, as opaque spots, medium-sized, not fused, position not fixed; arranged in 2 rows on carapace, in 1 row on scaphocerite and uropodal exopod; positioned close to central axis of scaphocerite and uropodal exopod. Arthrobranchs: both posterior lobe on somite XII and anterior lobe on somite XIII dendritic.

**Type species**: by present designation, *Robustosergia robusta* (Smith, 1882) [43].

**Type locality:** North Atlantic, off Martha's Vineyard, Massachusetts, U. S. Fish Commission Stations 893 and 952, 37^o^ 17′N, 73^o^ 21′W (USNM syntype); and 34^o^ 28′50″ N, 75^o^22′50″ W (MCZ syntype).

**Etymology**: after type species *R. robusta* (from the Latin ‘*robusta”* meaning strong, probably an allusion to the exterior which is more robust than in most other sergestids; plus the root *‘sergia’*).

**Species included**: *Robustosergia extenuata* (Burkenroad, 1940) [27], *Robustosergia regalis* (Gordon, 1939) [35], *Robustosergia robusta* (Smith, 1882) [44], and *Robustosergia vityazi* (Vereshchaka, 2000) [7].

*Scintillosergia* **gen.n.**

urn:lsid:zoobank.org:act:4580C71C-6638-4F8A-90DE-00EA9BD0C90C

**Diagnosis**: Integument firm, frontal margin of rostrum oblique, no supraorbital or hepatic teeth, hepatic protrusion forming barb. First segment of A I elongate, ≥1.5 times as long as 3d segment; distoventral end of 3rd segment in males without process; distal tooth of scaphocerite reaching or overreaching end of blade; maxilliped I developed, 3-4-segmented, maxilliped III<2.0 times as long as Cp, not dimorphic sexually, dactyl not subdivided into specialized subsegments; pereopods I–II ischia without strong movable spines and distally curved tooth; pereopod II with merus lacking protrusion and chela bearing equal fingers, without long setae; chela of pereopod III without strong curved spines and long setae on propodus, fingers subequal; pereopods IV–V present, 6-segmented; pereopod V setose along both margins; uropodal exopod with outer spine, proximal segment not setose along outer margin. Male clasping organ: developed, with 1–7 serrated bristles in an unordered heap, tubercle present. Petasma: LA well-developed, LC well-developed, entire, without pillow at base, twisted with LT, LI well-developed, straight, inflated, LT rudimentary, PU present, with hook, PV absent. Photophores: dermal, lens-bearing, small, position fixed; arranged in 2 rows on carapace, in 1 row on scaphocerite (7 organs) and uropodal exopod (2 organs on basal segment and 1 organ on distal segment); positioned close to central axis of scaphocerite and uropodal exopod. Arthrobranchs: both posterior lobe on somite XII and anterior lobe on somite XIII dendritic.

**Type species**: by monotypy, *Scintillosergia scintillans* (Burkenroad, 1940) [27].

**Type locality:** Southwestern Pacific, 25^o^54′S, 172^o^ 37′E.

**Etymology**: after type species *Sergia scintillans* (from the Latin ‘scintillans*”* meaning sparkling, an allusion to numerous photophores shining in live specimens, plus the root *‘sergia’*).

**Species included**: *Scintillosergia scintillans* (Burkenroad, 1940) [27].

*Sergestes* **H. Milne-Edwards, 1830**

**Diagnosis**: Integument firm, frontal margin of rostrum oblique, supraorbital tooth present, hepatic protrusion forming tooth. First segment of A I not elongate, <1.5 times as long as 3d segment; distoventral end of 3rd segment in males without process; distal tooth of scaphocerite reaching or overreaching end of blade; maxilliped I developed, 3-4-segmented, maxilliped III<2.0 times as long as Cp, not dimorphic sexually, dactyl subdivided into 6-7 specialized subsegments; pereopods I–II ischia without strong movable spines and distally curved tooth; pereopod II with merus lacking protrusion and chela bearing equal fingers, without long setae; chela of pereopod III without strong curved spines and without long setae on propodus, fixed finger subequal to dactyl; pereopods IV–V present, 6-segmented; pereopod V setose along one margin; uropodal exopod with outer spine, proximal segment not setose along outer margin. Male clasping organ: developed, without serrated bristles, tubercle present. Petasma: LA rudimentary, LC well-developed, divided, without pillow at base, twisted with LT, LI well-developed, straight, slender, LT well-developed, entire, PU present, without hook, PV rudimentary, triangle, without hooks, suckers, spines, or apical lashes. Photophores: organ of Pesta present, dermal organs absent. Arthrobranchs: posterior lobe on somite XII lamellar, anterior lobe on somite XIII dendritic.

**Type species**: By monotypy, *Sergestes atlanticus* H. MilneEdwards, 1830 [9].

**Type locality:** North Atlantic Ocean near Azores.

**Species included**: *Sergestes atlanticus* H. MilneEdwards, 1830 [9]

*Sergia* **Stimpson, 1860**

**Diagnosis**: Integument membranous, frontal margin of rostrum oblique, no supraorbital or hepatic teeth, hepatic protrusion inconspicuous. First segment of A I elongate, ≥1.5 times as long as 3d segment; distoventral end of 3rd segment in males without process; distal tooth of scaphocerite not reaching end of blade; maxilliped I developed, 3-4-segmented, maxilliped III<2.0 times as long as Cp, not dimorphic sexually, dactyl not subdivided into specialized subsegments; pereopods I–II ischia without strong movable spines and distally curved tooth; pereopod II with merus lacking protrusion and chela bearing equal fingers, without long setae; chela of pereopod III without strong curved spines and long setae on propodus, fingers subequal; pereopods IV–V present, 6-segmented; pereopod V setose along both margins; uropodal exopod with outer spine, proximal segment not setose along outer margin. Male clasping organ: developed, without serrated bristles, tubercle present. Petasma: LA well-developed, LC well-developed, entire, without pillow at base, not twisted with LT, LI well-developed, straight, slender, LT well-developed, entire, PU present, with hook, PV well-developed, entire, elongate, without hooks, suckers, spines, or apical lashes. Photophores: dermal photophores and organ of Pesta absent. Arthrobranchs: both posterior lobe on somite XII and anterior lobe on somite XIII dendritic.

**Type species**: by present designation, *Sergia tenuiremis* (Krøyer, 1855) [33].

**Type locality**: Tropical Atlantic, ca. 4.5°N, 21°W, coll. Hr. Fries (information from Danish introduction in [33])

**Species included:** *Sergia inoa* (Faxon, 1893) [41], *Sergia japonica* (Bate, 1881) [40], *Sergia laminata* (Burkenroad, 1940) [27], and *Sergia tenuiremis* (Krøyer, 1855) [33].

### 3. Key to Genera of the Family Sergestidae

1. Pereopods IV–V absent ……………***Acetes***

- Pereopods IV–V present ……………2

2. Pereopod IV with 6 segments……………3

- Pereopod IV with 5 or 7 segments……………18

3. Maximum height of rostrum at middle of its length ……………***Petalidium***

- Maximum height of rostrum near tip……………4

4. Organ of Pesta absent. Body opaque in live specimens, or, if semi-transparent, with dermal photophores……………5

- Organ of Pesta present. Body semi-transparent in live specimens, without dermal photophores……………12

5. Integument membranous, dermal photophores absent……………***Sergia***

- Integument firm, dermal photophores present……………6

6. Dermal photophores without lens, visible as opaque spots……………7

- Dermal photophores with lens……………9

7. Photophores as large, partly fused organs, arranged in 2 rows on scaphocerite and a triangular patch on uropodal exopod……………***Phorcosergia***

- Photophores small, not fused, arranged in 1 row on scaphocerite, and 1 row (randomly reduced to 1 organ) on uropodal exopod……………8

8. Ocular papilla developed (>0.3 times as long as wide). LC of petasma without pillow at base, not twisted with LT, LT entire. Photophores on uropodal exopod positioned close to inner margin……………***Gardinerosergia***

- Ocular papilla rudimentary (<0.3 times as long as wide). LC of petasma with pillow at base, twisted with LT, LT divided. Photophores on uropodal exopod positioned close to median line……………***Regalosergia***

9. Photophores: in 2 lateral rows on carapace, 7 or more organs on scaphocerite……………10

- Photophores: in a single lateral row on carapace, 6 or fewer organs on scaphocerite……………11

10. Photophores: 7 organs on scaphocerite, 2 organs on proximal segment and 1 on distal segment of uropodal exopod. Petasma: LC divided, LI inflated, LT rudimentary, PV absent……………***Scintillosergia***

- Photophores: 10–15 organs on scaphocerite, 4–8 organs on proximal segment and 3–5 on distal segment of uropodal exopod. Petasma: LC entire, LI slender, LT well-developed, PV present……………***Prehensilosergia***

11. Photophores: 4–6 organs both on lateral carapace row and on scaphocerite. Petasma: PV without hooks and suckers……………***Challengerosergia***

- Photophores: 2–3 organs both on lateral carapace row and on scaphocerite. Petasma: PV with hooks and suckers……………***Lucensosergia***

12. Outer margin of uropodal exopod with tooth, not setose along proximal segment (proximal to the tooth)……………13

- Outer margin of uropodal exopod without tooth, setose at least along part of proximal segment.……………15

13. First segment of antennule elongate, ≥1.5 times as long as 3rd segment, distal tooth of scaphocerite not overreaching blade, maxilliped III sexually dimorphic. Petasma: PU with hook, PV with simple spines. Arthrobranch: posterior lobe on segment XII (above pereopod III) dendritic……………***Eusergestes***

- First segment of antennule not elongate, <1.5 times as long as 3rd segment, distal tooth of scaphocerite overreaching blade, maxilliped III sexually not dimorphic. Petasma: PU without hook, PV unarmed. Arthrobranch: posterior lobe on segment XII (above pereopod III) lamellar……………14

14. Rostrum triangular, not reaching middle of eyestalk. Endopod of maxilliped I with 3 segments. Petasma: LA rudimentary, LC developed, divided, PV present……………***Sergestes***

- Rostrum elongate, much overreaching middle of eyestalk. Endopod of maxilliped I with 2 segments. Petasma: LA developed, LC rudimentary, PV absent……………***Cornutosergestes***

15. Maxilliped III moderately elongated, <2.0 times as long as carapace; chela of pereopod II with very long setae. Arthrobranch: posterior lobe on segment XII (above pereopod III) dendritic……………***Deosergestes***

- Maxilliped III much elongated,>2.0 times as long as carapace; chela of pereopod II without very long setae. Arthrobranch: posterior lobe on segment XII (above pereopod III) lamellar ……………16

16. Rostrum with vertical frontal margin and beak-like terminal tooth, ocular papilla prominent, distal tooth of scaphocerite not overreaching blade, maxilliped III>2.8 times as long as carapace, pereopod II without distally curved hooks on ischium, without protrusion on merus; chela with unequal fingers, pereopod III with strong curved spines proximal to tufts of long setae on propodus, pereopod V with distal segment setose along both margins. Petasma: LC absent, LI rudimentary, slender, PU with hook, PV developed……………***Allosergestes***

- Rostrum with oblique frontal margin, no beak-like terminal tooth, ocular papilla uncertain, distal tooth of scaphocerite much overreaching blade, maxilliped III 2.0–2.8 times as long as carapace, pereopod II with distally curved hooks on ischium and protrusion on merus; chela with subequal fingers, pereopod III without strong curved spines proximal to tufts of long setae on propodus, pereopod V with distal segment setose along one margin. Petasma: LC present, LI developed, inflated, PU without hook, PV rudimentary……………17

17. Maxilliped III dactyl subdivided into 4 specialized subsegments, pereopod I with strong movable spines on ischium, outer margin of uropodal exopod setose partly……………***Parasergestes***

- Maxilliped III dactyl subdivided into 6 specialized subsegments, pereopod I without strong movable spines on ischium, outer margin of uropodal exopod setose entirely ……………***Neosergestes***

18. Pereopod IV with 5 segments……………***Peisos***

- Pereopod IV with 7 segments……………***Sicyonella***

### 4. Phylogenetic Remarks and New Taxonomy

The phylogeny of the former genera *Sergestes* and *Sergia* presented here is the result of simultaneous use of all available morphological characters. There are three groups of characters relating to (1) general decapod morphology, (2) morphology of the male copulatory organs, and (3) morphology of the photophores. Previous attempts to classify sergestid shrimps have mainly focused at one of these character systems resulting in a lack of consensus concerning sergestid systematics.

Table 4 shows that only five genera are supported by one type of synapomorphies: either general morphological (*Sergia*) or sex-related (*Sergestes, Robustosergia, Challengerosergia, Scintillosergia).* Most genera are supported by a combination of synapomorphies: general morphological and sex-related (*Eusergestes, Cornutosergestes, Neosergestes, Parasergestes, Deosergestes, Allosergestes*), general morphological and photophore-related (*Gardinerosergia*), or sex- and photophore-related (*Phorcosergia, Prehensilosergia, Lucensosergia*). Use of one type of the characters would apparently not be resulted in a satisfactorily resolved tree. Thus, a simultaneous use of a broad suite of characters including sex- and photophore-related characters is necessary for any successful attempt of sergestid phylogeny.

Our analysis does not support the clades *Sergia* and *Sergestes* as recognized previously [4], [11]. Instead, 15 separate genera within these former taxa are supported by synapomorphies and Bootstrap analysis. ‘*Sergia’* and ‘*Sergestes’* groups were suggested early, although various experts proposed different taxonomic statuses for them. Initially, the status was generic (first descriptions by Milne Edwards [9] and Stimpson [10]), but later both genera were combined in the single genus *Sergestes* [4], [11]. Burkenroad [23], [27] suggested a subdivision of the genus into the two subgenera *Sergestes* s.s. and *Sergia*, based on differences in types of photophores and pigmentation, which was later defined formally by Yaldwyn [5]. The taxonomic status of both subgenera was raised to generic level by Omori [6]. Omori's classification was subsequently used by most authors but some hinted at the possibility that both genera might have higher taxonomic status, and that the species groups within each genus might deserve the status of valid genera [7] – [8].

Considering the twisted taxonomy of the ‘*Sergia-Sergestes’* group, Vereshchaka [7] – [8] underlined that development of a new classification should be postponed until revisions of the world fauna had been completed and phylogenetic analyses based on a broad set of characters had been undertaken. The situation became more complicated when Judkins, Kensley [18] offered only very brief diagnoses for several new genera. However, the results of the phylogenetic analysis in this work indicate that most of their new genera are valid. In the present work we recognise that Judkins and Kensley's genus ‘*Sergestes*’ contains two valid genera (*Sergestes* Milne-Edwards, 1830 [9] and *Cornutosergestes* n.gen.) and that the genus *Sergia* s.s. consists of eight genera (see fig. 8 and Table 2).

### 5. Vertical Distribution of Clades

**Figure 9:**
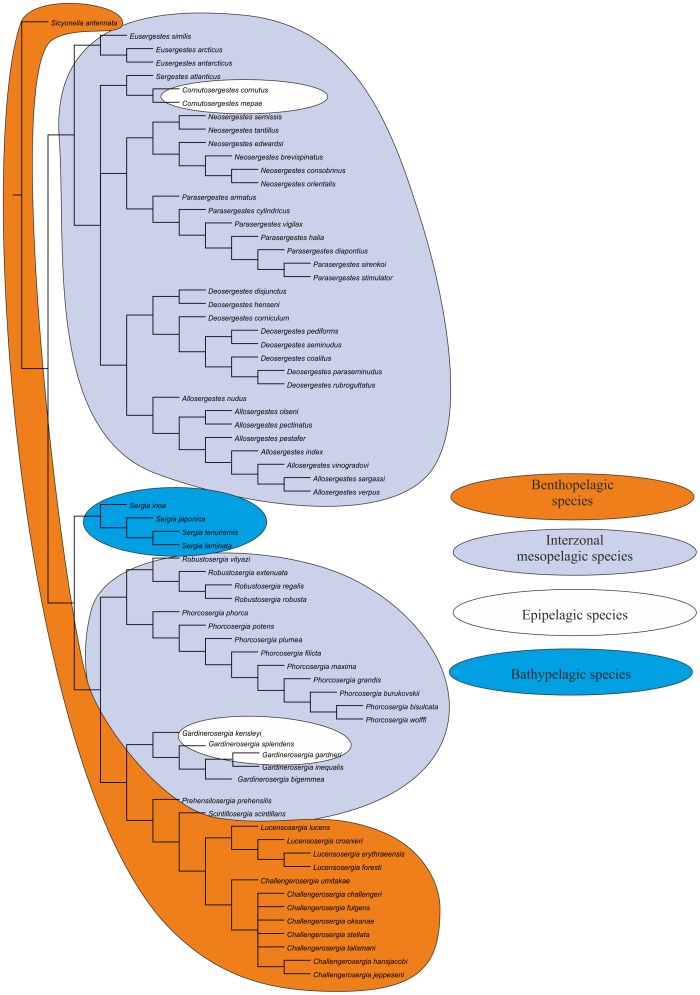
Distribution of clades within principal oceanic biotopes.

Several evolutionary patterns relating to vertical distribution (benthic, pelagic, etc.) of the genera under consideration can be elucidated based on the consensus tree presented here (Fig. 9). *Sicyonella antennata,* used as outgroup in the present analysis, is benthopelagic (Fig. 9, brown). Since other related sergestid taxa (*Acetes, Peisos*) are also benthopelagic, this optimises as the likely original habitat for sergestids. The genera *Lucensosergia* and *Challengerosergia* are characteristic of near-bottom layers above seamounts, continental slopes, and shelves. Most benthopelagic species have local ranges [7] – [8], [45] in the Atlantic, Indian, and Pacific oceans living above shelves, continental slopes, and seamounts. Our phylogeny shows that several lineages have penetrated into the pelagic realm independently from a near-bottom origin. Most clades and genera are typical interzonal migrants living in the low mesopelagic zone (depths 400–800 m) in the daytime and ascending to the epipelagic zone (100–200 m) at night (Fig. 9, light blue). These are species with regional geographic ranges, occupying temperate and/or tropical zones and including the genera *Eusergestes, Sergestes, Neosergestes, Parasergestes, Allosergestes, Robustosergia*, *Phorcosergia,* and parts of *Gardinerosergia*.

Some species of the genera have become epipelagic living in the upper 200 m (Fig. 9, transparent). These are *Cornutosergestes* and some species of *Gardinerosergia.* Conversely, *Sergia* went to the deep bathypelagic zone (800–3000 m deep Fig. 9, blue); this genus shows typical adaptations to deep-sea living such as high fat-content, membranous integument, small cornea, absence of photophores, etc.

### 6. Geographic Distribution

**Figure 10:**
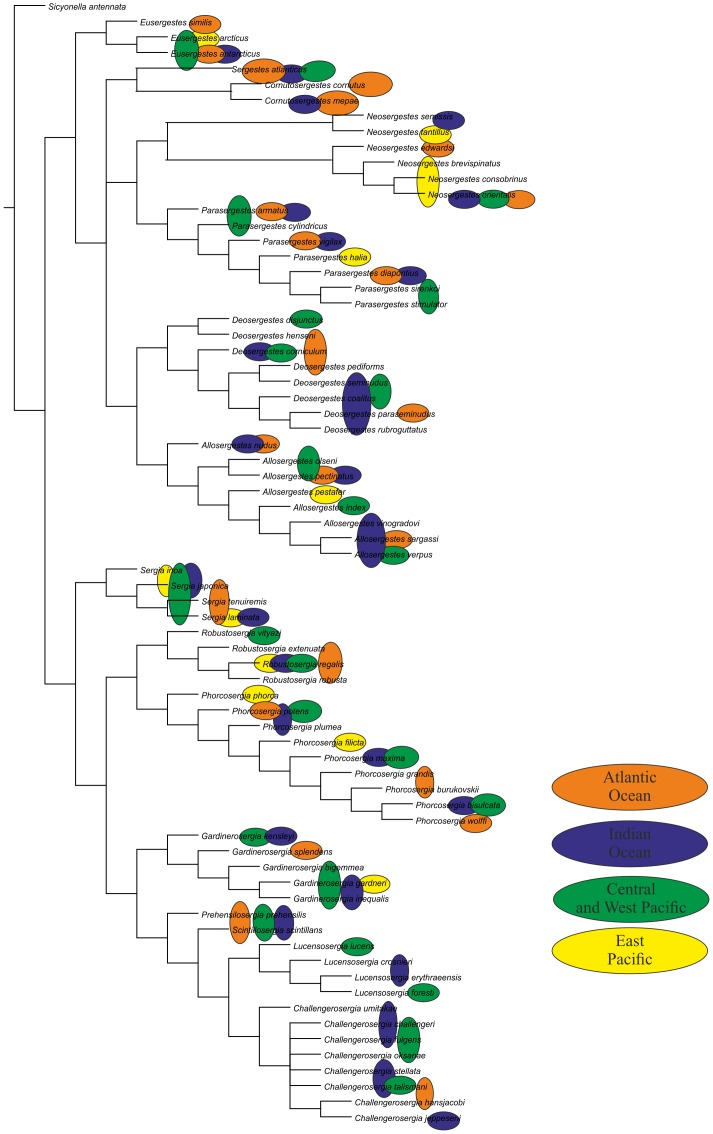
Geographical distribution of clades.

The geographic distribution of the species is mapped on the strict consensus tree in Fig. 10. There is no simple relation between presented phylogeny and the species distribution, so any conclusions concerning ‘centres of origin’ are very difficult to obtain. Numbers of species occurring in different oceans are similar: 32 in the Atlantic, 37 in the Indian Ocean, and 38 species in the Central and West Pacific. East Pacific is inhabited by a relatively low number of species (14) that may be related to oxygen-depleted conditions recorded in many areas of this region.

The overall pattern of geographic distribution broadly indicates that speciation has occurred mainly in the tropical and subtropical waters of all oceans. Indeed, 68 of 71 recorded species are found in the tropical/subtropical waters of the World Ocean and only the genus *Eusergestes* inhabits temperate/subpolar waters.

One general pattern concerning distribution is that the species ranges of most species within most genera (except epipelagic *Cornutosergestes* and bathypelagic *Sergia*) are restricted to a single ocean (Table 5). These genera are meso- or bathypelagic that indicates importance of sympatric speciation [46] – [47] within these zones. This rule is even more pronounced with respect to benthopelagic species having local species ranges, as is often seen for non-pelagic marine taxa.

**Table 5:** Species ranges of considered genera.

Genera	Species ranges			Remarks
	Panoceanic	Two oceans	One ocean	
***Eusergestes***	*antarcticus*		*arcticus*	mesopelagic
			*arcticus*	
***Sergestes***	*atlanticus*			mesopelagic
***Cornutosergestes***		*cornutus*		epipelagic
		*mepae*		
***Neosergestes***	*orientalis*		*semissis*	mesopelagic
			*tantillus*	
			*brevispinatus*	
			*consobrinus*	
			*edwardsii*	
***Parasergestes***	*armatus*	*diapontius*	*stimulator*	mesopelagic
		*vigilax*	*cylindricus*	
			*halia*	
			*sirenkoi*	
***Deosergestes***	*coalitus*	*corniculum*	*disjunctus*	mesopelagic
		*seminudus*	*henseni*	
		*paraseminudus*	*pediformis*	
			*rubroguttatus*	
***Allosergestes***	*pectinatus*	*nudus*	*oleseni*	mesopelagic
		*sargassi*	*pestafer*	
		*verpus*	*index*	
			*vinogradovi*	
***Sergia***	*laminata*	*tenuiremis*		bathypelagic
	*japonica*	*inoa*		
				
***Robustosergia***	*regalis*		*extenuata*	mesopelagic
			*robusta*	
			*vityazi*	
				
***Gardinerosergia***	*gardineri*	*kensleyi*	*bigemmea*	mesopelagic
		*inequalis*	*splendens*	
***Phorcosergia***	*potens*	*maxima*	*burukovskii*	mesopelagic
		*bisulcata*	*filicta*	
			*grandis*	
			*phorca*	
			*plumea*	
			*wolffi*	
***Prehensilosergia***	*prehensilis*			mesopelagic
***Scintillosergia***	*scintillans*			mesopelagic
***Lucensosergia***			*crosnieri*	benthopelagic
			*erythraeensis*	
			*foresti*	
			*lucens*	
***Challengerosergia***	*talismani*	*challengeri*	*stellata*	benthopelagic
		*fulgens*	*umitakae*	
			*hansjabobi*	
			*jeppeseni*	
			*oksanae*	

One interesting pattern is that most often a single species with panoceanic distribution can be found within each genus (Table 5). However, this does not apply to the epipelagic genus *Cornutosergestes*, perhaps because the surface anticyclonic gyres in this region result in geographic isolation, thereby preventing wider distribution of shallow-living species. Also, such a pattern does not apply to the bathypelagic species of *Sergia,* almost all of which are panoceanic probably being distributed by the Great Ocean Conveyor current.

The species with panoceanic distributions are of special interest for molecular studies, since, despite the morphological similarity between populations, cryptic speciation may be involved [48]. If, on the other hand, the panoceanic species are not distinguishable genetically, it would be a challenge to explain their distribution in more detail than we have here.

### 7. The Photophores

**Figure 11:**
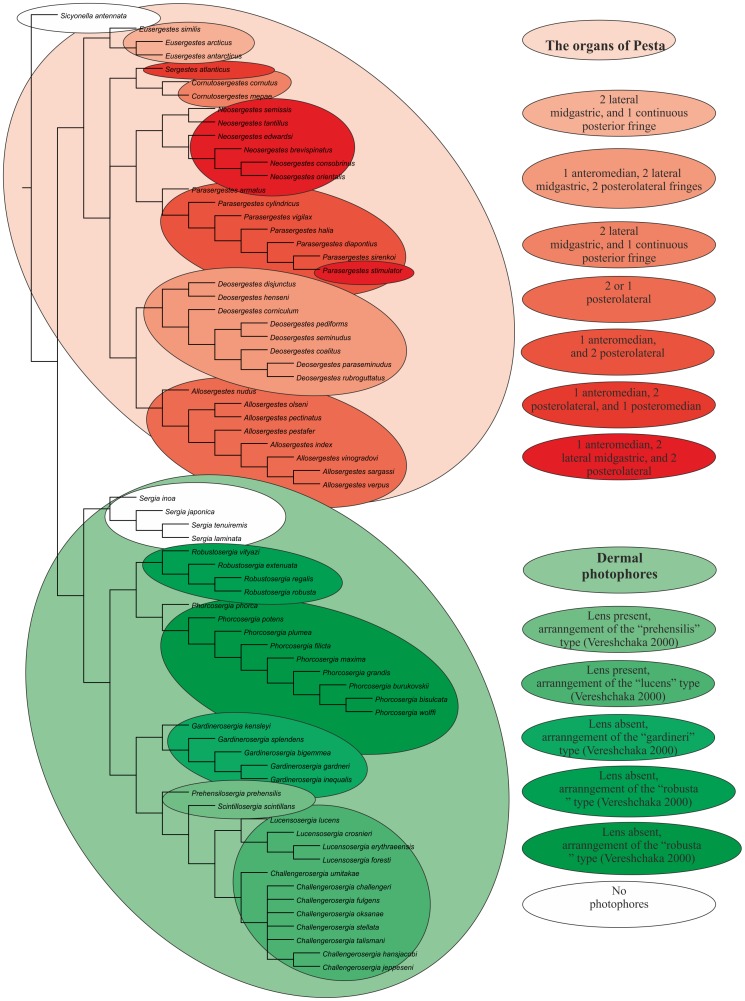
Distribution of photophore types in the clades.

Most species included in the phylogenetic analysis possess luminescent organs. Species of the genera *Eusergestes, Sergestes, Cornutosergestes, Neosergestes, Parasergestes, Allosergestes,* and *Deosergestes* have the organ of Pesta (Fig. 11, in pink), which is a lluminescent modified area of the gastrohepatic glands found within the cephalothorax. The morphology and histology of this organ are different in each of the generic clades (details in [8]) and are therefore of phylogenetic importance.

The genera *Prehensilosergia, Scintillosergia, Lucensosergia, Challengerosergia, Gardinerosergia, Robustosergia,* and *Phorcosergia* have dermal photophores (Fig. 11, in green), which are either lens-bearing or lens-less. Lens-bearing photophores may be arranged in two different ways (details in [8]) and are characteristic of the genera *Prehensilosergia, Scintillosergia, Lucensosergia,* and *Challengerosergia*. Lens-less photophores (visible in preserved material as opaque spots) are arranged in three different types, each of which is characteristic of *Gardinerosergia, Robustosergia,* or *Phorcosergia*.

Sergestid dermal photophores are directed downwards and are important for countershading [49] – [50]. In the bathypelagic zone, countershading is ineffective and all species of the deep-sea genus *Sergia* have no photophores.

### 8. Behavioral Strategies: Offensive *Versus* Protective

The behaviour of sergestid species is not well known because most of them are mesopelagic and difficult to observe. However, visual observations were available for *Lucensosergia lucens* [51], *Eusergestes similis* [52], and the closely related *Acetes sibogae* [53]. The data indicate that the shrimps capture prey by combined actions of the first three pairs of pereopods and the third maxillipeds before transferring it to the more dorsal second maxillipeds. Simultaneously they move using the pleopods (forward movement) or uropods (escape backwards). The morphology of these appendages reflects the presence of two fundamentally different behavioral strategies: offensive and protective. Some taxa feed on live planktonic animals and have developed a set of structures relating to this feeding mode; this is termed here an ‘offensive strategy’. On the other hand, sergestids are themselves preyed upon by larger carnivores like squids and fishes and have developed a set of characters related to protective/avoidance behavior; this is termed a ‘protective strategy’.

**Figure 12:**
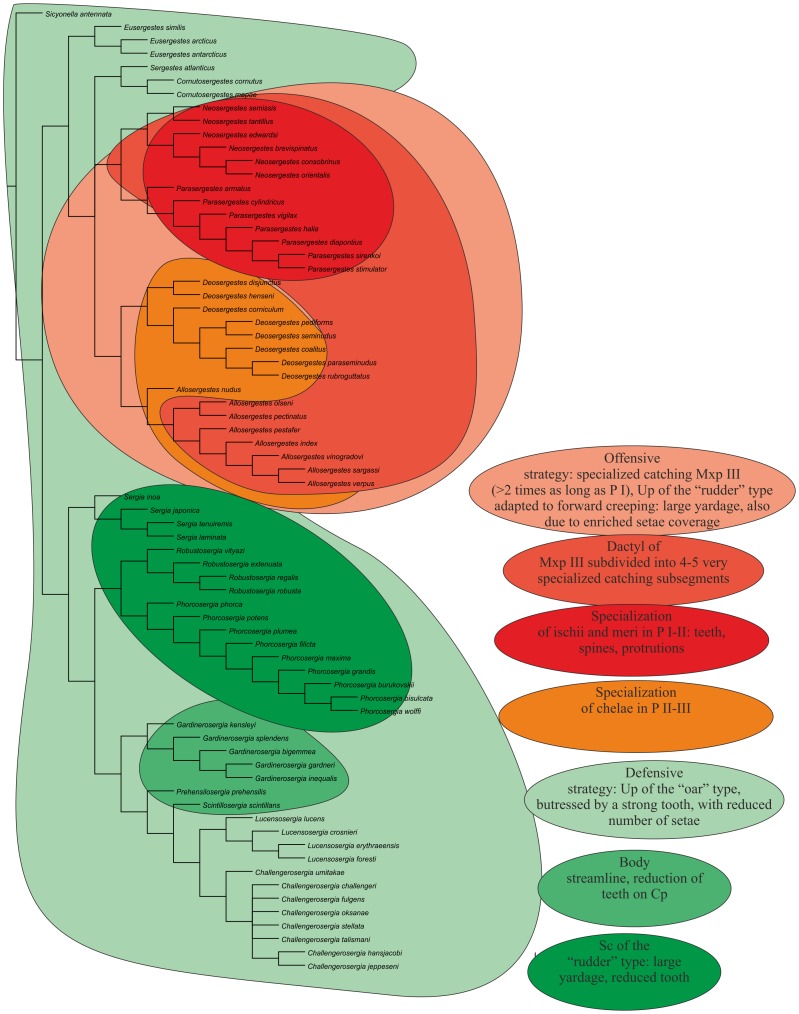
Distribution of selected characters in the clades.

Several genera (*Neosergestes, Parasergestes, Allosergestes,* and *Deosergestes*) show a set of characters related to the offensive strategy (Fig. 12, red spectrum): (1) they have much enlarged maxillipeds (>2 times as long as first pereopods), which are stretched forward to catch the prey; (2) their uropods may act as rudders (an increased surface area of the blade, which is also enhanced by greater setal coverage), during their slow swimming forward towards prey using the pleopods. Further morphological specializations for feeding on other planktonic animals (offensive strategy) are seen within the genera *Neosergestes, Parasergestes,* and *Allosergestes* (Fig. 12, light red). In these species the dactyl of maxilliped III is subdivided into 4–5 very specialized subsegments specialized for catching prey. The genera *Neosergestes* and *Parasergestes* (Fig. 12, red) have additional specialized structures for catching prey. The ischia and meri of pereopods I–II in these genera have various teeth, spines, and protrusions that may be used for the retention of prey. Specializations are also present in the genera *Allosergestes* and *Deosergestes* (Fig. 12, orange) which seem to be related to an offensive strategy; the chelae of pereopods I–II bear strong teeth and/or elongated setae, which may replace the dactyl functionally.

The remaining clades (the genera Eusergestes, Sergestes, Cornutosergestes, Prehensilosergia, Scintillosergia, Lucensosergia, Challengerosergia, Gardinerosergia, Robustosergia, Phorcosergia, and Sergia) show specializations relating to the protective strategy (escaping predators) (Fig. 12, the green spectrum). They have uropods that are buttressed by a strong tooth and with a reduced number of setae. These uropods are efficient as oars when the shrimp jumps backwards to escape predators. A streamlined body lacking protruding teeth or spines (e.g., hepatic or supraorbital spines) would aid in their escape from predators. Such a morphology is found in the clade which includes Prehensilosergia, Scintillosergia, Gardinerosergia, Robustosergia, Phorcosergia, and Sergia (Fig. 12, green).

Finally, the genera *Robustosergia, Phorcosergia,* and *Sergia* (Fig. 12, dark green) show a further advance in the development of a protective strategy. Here, the large size and dense setal coverage of the scaphocerites (lateral blades of antennae) suggest they act as rudders during backward jumps generated by the use of the oar-type uropods.

Both the protective and offensive strategies are also related to swarming behavior. All commercially important species (*Lucensosergia lucens*, *Eusergestes similes, Eusergestes arcticus*) belong to the “protective strategy” group where avoidance of predators is favoured. All species of this group (except for a few rare ones) are numerous in plankton samples, which suggests they aggregation in shoals. Indeed, the maximum concentration observed via underwater camera in aggregations of *L. lucens* may reach 6 ind/m^3^ [54]. The species that favour offensive strategies are regularly recorded but are always few in numbers, which suggests that shoaling behavior is not the case here. Instead, their relative rarity, small size, and remarkable transparency may protect these species from carnivores and thereby explain why no protective morphological structures are present.

## Conclusions

There are always subjective aspects of basing a classification on a phylogeny. The most important point is that any suggestion should always be based on a phylogeny using as much data as possible. We tried both to use results of our analysis of three sets of characters and to keep conservative approach as much as we could: wherever possible, we did not erect new taxa and did not confuse sergestid taxonomy any more. All recognized genera are monophyletic, supported by synapomorphies and significant Bootstrap values. Our analysis does not support the genera *Sergia* and *Sergestes* sensu Hansen and Omori; instead, it confirms validity of 6 previously established genera [18] and supports 8 new genera. Future work, for example involving molecular data (now very scant in the Gene Bank), will test the validity of suggested phylogeny and thereby the classification. Phylogenetic analysis of the other sergestid genera *Acetes, Petalidium, Sicyonella, Peisos* may also help in better understanding of the sergestid phylogeny.

Our studies revealed the following phenomena:

1. independent phylogenies of 3 sets of characters (general decapod morphology, male copulatory organs, and photophores);
2. existence of two prominent strategies, protective and offensive, expressed both in morphology and behaviour at generic level;
3. biogeographical rule for mesopelagic genera “a single panoceanic species – most species with local distribution”. Molecular studies on these panoceanic species may help to understand whether they really represent a single species-level taxon or they are conglomerates of two or more species.

Further studies will show how widely these phenomena are spread among other taxonomic groups.
